# A chromosomal-scale genome assembly of modern cultivated hybrid sugarcane provides insights into origination and evolution

**DOI:** 10.1038/s41467-024-47390-6

**Published:** 2024-04-08

**Authors:** Yixue Bao, Qing Zhang, Jiangfeng Huang, Shengcheng Zhang, Wei Yao, Zehuai Yu, Zuhu Deng, Jiaxin Yu, Weilong Kong, Xikai Yu, Shan Lu, Yibin Wang, Ru Li, Yuhan Song, Chengwu Zou, Yuzhi Xu, Zongling Liu, Fan Yu, Jiaming Song, Youzong Huang, Jisen Zhang, Haifeng Wang, Baoshan Chen, Xingtan Zhang, Muqing Zhang

**Affiliations:** 1 State Key Laboratory for Conservation and Utilization of Subtropical Agri-Biological Resources & Guangxi Key Laboratory for Sugarcane Biology, Guangxi University, Nanning, 530005 China; 2 National Key Laboratory for Tropical Crop Breeding, Shenzhen Branch, Guangdong Laboratory for Lingnan Modern Agriculture, Genome Analysis Laboratory of the Ministry of Agriculture, Agricultural Genomics Institute at Shenzhen, Chinese Academy of Agricultural Sciences, Shenzhen, Guangzhou 518120 China

**Keywords:** Agricultural genetics, Comparative genomics, Evolutionary genetics, Plant breeding

## Abstract

Sugarcane is a vital crop with significant economic and industrial value. However, the cultivated sugarcane’s ultra-complex genome still needs to be resolved due to its high ploidy and extensive recombination between the two subgenomes. Here, we generate a chromosomal-scale, haplotype-resolved genome assembly for a hybrid sugarcane cultivar ZZ1. This assembly contains 10.4 Gb genomic sequences and 68,509 annotated genes with defined alleles in two sub-genomes distributed in 99 original and 15 recombined chromosomes. RNA-seq data analysis shows that sugar accumulation-associated gene families have been primarily expanded from the ZZSO subgenome. However, genes responding to pokkah boeng disease susceptibility have been derived dominantly from the ZZSS subgenome. The region harboring the possible smut resistance genes has expanded significantly. Among them, the expansion of WAK and FLS2 families is proposed to have occurred during the breeding of ZZ1. Our findings provide insights into the complex genome of hybrid sugarcane cultivars and pave the way for future genomics and molecular breeding studies in sugarcane.

## Abstract (web summary)

Modern sugarcane cultivars have complicated genome due to interspecific crosses and multiple backcrossing. Here, the authors report the haplotype-resolved, chromosome-level genome assembly of a modern hybrid sugarcane cultivar and reveal the expansion of genes related to sugar accumulation and smut resistance.

## Introduction

Sugarcane, a member of the sub-tribe *Saccharinae* within the *Andropogoneae* tribe^1^, is arguably the most voluminous crop grown worldwide, with its weight surpassing that of staple food crops such as rice, maize, or wheat (http://www.fao.org/faostat/en/#home). As the global leader in sugar production and a prime candidate for bioenergy production, sugarcane accounts for over 80% of the world’s sugar and 40% of bioethanol yield, which gives it an estimated annual economic value of up to US $90 billion (https://www.fao.org/faostat/zh/#data/QV).

Modern sugarcane hybrids have their origins in interspecific crosses between the thick-stalked, high-sugar *Saccharum officinarum* and the wild, thin-stalked, low-sugar *Saccharum spontaneum*, with additional multiple backcrossing with *S. officinarum*^2,3^. This intricate hybridization process not only enhanced the vigor, robustness, tillering, disease resistance, and environmental adaptability of modern cultivated sugarcane hybrids but also escalated the complexity of their genome beyond that of their progenitors^4^. The resulting hybrid genome comprises a mix of aneuploid and homo(eo)logous chromosomes unevenly inherited from the two polyploid progenitor species, leading to a large genome size of ~10 Gb. The number of chromosomes in hybrids can range from 100 to 130 depending on the specific cross^5,6^. Approximately 70 to 80% of these chromosomes are derived from *S. officinarum*, 10 to 20% from *S. spontaneum*, and about 10% result from interspecific recombination^4,7–9^.

While decoding the auto-polyploid genomes of *S. officinarum* and *S. spontaneum*^10,11^ has somewhat clarified the challenges of polyploid phasing through technological advancements and algorithmic innovations^12^, assembling the sugarcane hybrid genome presents an unprecedented challenge due to its heterologous aneuploid nature with gene loci consisting of 8–14 homo(eo)logous copies^13–15^. Published hybrid sugarcane genomes such as SP80-3280^16^, KK3^17^, and R570^18^ only partially represent the complete information of hybrid sugarcane cultivars due to incomplete assembly. In addition, one of the most severe diseases, smut, caused 20 to 30% yield and sugar losses in China. Pokkah boeng disease (PBD), a century-old disease worldwide, is becoming more severe in China due to the overuse of nitrogen fertilizer and the warming of the climate.

This work presents a haplotype-resolved and chromosome-level de novo genome assembly of the hybrid sugarcane cultivar ZZ1. We reveal the expansion of genes related to sugar accumulation, smut resistance, and the origin of genes associated with PBD susceptibility.

## Results

### Karyotype and genome assembly

**Fig. 1:**
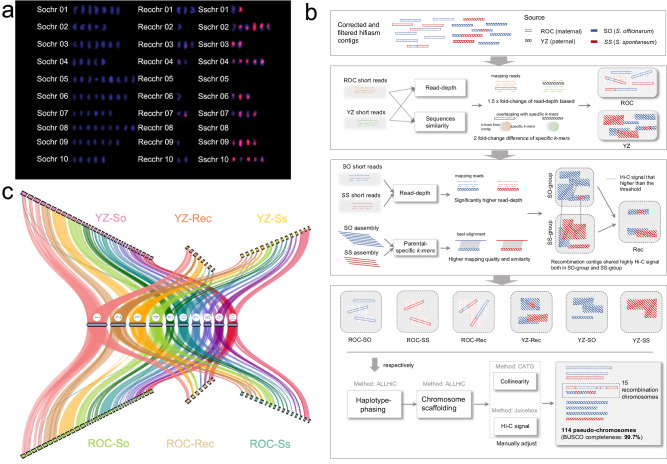
Karyotype analysis and chromosome-scale assembly of the modern hybrid sugarcane (ZZ1) genome. **a** Karyotype analysis, chromosomes with different ancestors of SO (left), SS (right), and recombination between SO and SS (mid) were shown. **b** overview of sequencing and assembly strategy: (I) assembly, filter, and correct contigs, (II) separate contigs into ROC and YZ groups based on specific *k*-mer difference, (III) separate contigs into SO and SS groups with mapping quality and similarity, contigs from Rec chromosomes are collected based on Hi-C signals between SO- and SS-derived contigs, (IV) apply haplotype phasing and chromosome-scaffolding in all six groups respectively and Manually adjust. **c** genomic features of the ZZ1 genome, the reference genome at the center position is Sorghum (Sb), and the collinearity among six chromosome groups in (**b**) with Sb were drawn respectively.

The modern hybrid sugarcane cultivar originated from hybridization between two ancestral *Saccharum* species, namely *S. spontaneum* and *S. officinarum*, followed by several rounds of backcross with *S. officinarum*. The high ploidy and extensive recombination between the two subgenomes make the cultivated sugarcane an ultra-complex genome that still needs to be resolved. We initially investigated the genome features of the modern hybrid sugarcane cultivar ZZ1 using flow cytometry and karyotype analysis. The estimated genome size is ~9.0 Gb (Table 1), consistent with previous estimations of modern hybrid sugarcane cultivars^18^. Karyotype analysis using the Oligo probes designed from *S. spontaneum* specific abundant retrotransposons identified a total of 114 chromosomes, of which 68 originated from *S. officinarum* (So), 31 from *S. spontaneum* (Ss), and 15 represented recombination (Rec) between subgenomes (Fig. 1a). Chromosome painting with chromosome-specific probes designed from the *S. officinarum* (LA-purple) monoploid assembly classified these 114 chromosomes into ten homeologous groups, following the same naming rule in the R570^18^ (Supplementary Table 1).

**Table 1:** Statistics of genome assembly and annotation

Sequencing	Modern hybrid sugarcane
**PacBio Sequel II HiFi sequencing**	
clean data (Gb)	325
Sequencing depth (×)	32.5
Average reads length (bp)	13,145
Reads N50 (bp)	13,219
** Hi-C sequencing**	
Clean data (Gb)	1175
Sequencing depth (×)	117.5
**Chromosomal-level genome assembly and annotation**	
Estimated genome size (Gb)/2 C	9.0
Assembly size (Gb)	10.4
% of estimated genome size	115.6
Scaffold N50 (Mb)	81.0
BUSCO completeness of assembly (%)	99.7
Total number of genes/alleles	370,103
BUSCO completeness of annotation (%)	99.0

To solve the assembly of this ultra-complex genome, we incorporated multiple sequencing technologies, including Illumina, PacBio CCS platforms, and proximity ligation approach, generating 656 Gb short, 325 Gb high-fidelity (HiFi) long, and 1.175 Tb high-throughput chromatin conformation capture (Hi-C) short reads, respectively (Table 1 and Supplementary Tables 2, 3). To facilitate chromosome phasing, we also sequenced ~50× Illumina short reads for its two parental genomes (ROC25 and YZ89-7; Supplementary Fig. 1). The initial contigs were assembled using a widely applied HiFi assembler (i.e., hifiasm), resulting in 11.2 Gb sequences with the N50 of 527 kb (Supplementary Table 4). Those misjoined contigs were further identified based on abnormal chromatin interaction signals detected by Hi-C linked reads, refining 3.6% (5125/141,261) of total contigs. In addition, we identified 2.53 Mb artifactual sequences based on a whole-genome survey of artifactual *k*-mers and 835.53 Mb contamination, including organelle genomes and bacterial sequences. Removing these artifactual and contaminated sequences produces a high-quality contig-level assembly containing 10.4 Gb genomic sequences (Supplementary Table 4).

Pre-assigning contigs facilitated the scaffolding of this highly complex genome to different origins (see Methods; Fig. 1b). We first assigned these refined contigs to maternal (ROC) and paternal (YZ) groups based on resequenced parental genomes (ROC25 and YZ89-7). The two groups contain similar sequences (4.3 Gb in ROC and 4.7 Gb in YZ), accounting for 87% of the assembled size (Supplementary Table 5). We also traced the ancestral origination of assembled contigs by investigating the biased sequencing depth and similarity using published genomes and resequencing data of *S. spontaneum* (Ss) and *S. officinarum* (So), which classified 58.7% (6.1 Gb/10.4 Gb) sequences into So group, and Ss-derived sequences account for 23.1% (2.4 Gb/10.4 Gb) of the assembled genome, highly consistent with the previous estimation^19^. Our karyotype analysis revealed frequent chromosome recombination (Rec) between the two ancestral subgenomes, and the recombined sequences were further identified by detecting significantly high Hi-C contact signals between So- and Ss-derived contigs (see Methods). It shows that 1905 Mb sequences contribute to the 15 Rec chromosomes. Chromosome phasing and scaffolding were independently applied on the six pre-assigned groups (i.e., ROC-So, ROC-Ss, ROC-Rec, YZ-So, YZ-Ss, and YZ-Rec) using 59,906,451 (77.66%) lib valid paired-end Hi-C reads (Supplementary Table 3 and Supplementary Fig. 2), resulting in 87% (9.1/10.4 Gb) sequences anchored onto 114 pseudo-chromosomes, which represents a significant increase compared to the incompletely assembled genomes of previously published sugarcane cultivars^16–18^ (Supplementary Tables 5–7).

The quality of genome assembly was accessed using a series of approaches. Assessment using 1614 benchmarking universal single-copy orthologs (BUSCOs) showed that 99.7% of genes were completely recalled with 98.5% duplication (Supplementary Table 8). Alignment of 3.2 billion Illumina clean reads against the genome identified that all the genomic regions could be covered by at least five reads with a mapping rate of 99.94% (Supplementary Table 9). The synteny analysis revealed that most genes were ordered consistently with Sorghum and other published sugarcane genome annotation^10,11,16–18^ (Fig. 1c and Supplementary Fig. 3). The chromatin contact heatmap showed that the genomic sequences were well-organized along with the diagonals (Supplementary Figs. 2, 4). The LTR assembly index (LAI) calculation indicates that the genome assembly has a value of 12.27, qualifying it as a reference genome^20^.

### Annotation and genomic features

We annotated a total of 6.9 Gb repetitive sequences, accounting for 66.54% of the assembled ZZ1 genome, among which 36.45% repetitive sequences are annotated in So, 10.84% from Ss and 11.15% from Rec chromosomes (Supplementary Table 10). As the majority type of repetitive sequences, the long terminal repeat (LTR) retrotransposons account for 45.63% of the assembled genome (Supplementary Table 10 and Supplementary Fig. 5), which is similar to the ratio in the published sugarcane and sorghum genomes, ~41% in *S. spontaneum* (Np-X)^11^ and ~54% Sorghum^21^. The Kimura divergence calculation indicated that Ty1/Copia elements dominated recent transposon expansion events despite their lower frequency (15.65%) compared to Ty3/Gypsy elements (27.42%) throughout the genome (Supplementary Fig. 5).

We sequenced RNA samples from the ZZ1 tissues to annotate protein-coding genes. A total of 370,103 protein-coding genes, spanning a combined length of 1235.86 Mb, were annotated by protein-homology-predicted and RNA-seq-aligned methods, of which 92.14% (341,040) could be successfully validated by RNA-seq reads. Out of all the annotated genes, 30.03% (111,167) consisted solely of a single exon, while 14.26% (52,797) and 15.75% (58,308) genes had 5′UTR and 3′UTR annotations. The comprehensive approach to identifying allele genes within this ultra-complex genome relied on the monoploid genome-annotated gene sets from two sugarcane foundation species as reference (see Methods). After two rounds of protein alignment, we identified a total of 68,509 well-annotated genes with defined alleles into three subgenome groups (36,439 in the So subgenome, 27,760 in the Ss subgenome, and 4310 in the Rec chromosomes) (Supplementary Data 1). This annotation contained 26,650 (38.9%) genes with a single allele, 13,493 (19.7%) with two alleles, 9431 (13.8%) with three alleles, and 10.3, 7.6, 5.2, 2.8, 1.2, 0.4, and 0.1% with four to ten alleles, respectively. In addition, we identified 42 genes with 11 alleles and 20 genes with 12 allele counts. We further analyzed the homology of genes between two major subgenomes (So and Ss) in the ZZ1 genome. The statistical results showed that 23,670 genes were identified as homoeologous gene pairs of So-Ss subgenomes, while 12,769 and 3580 were detected as specific genes in the So and Ss, respectively (Supplementary Table 11).

### The pedigree identification and analysis

**Fig. 2:**
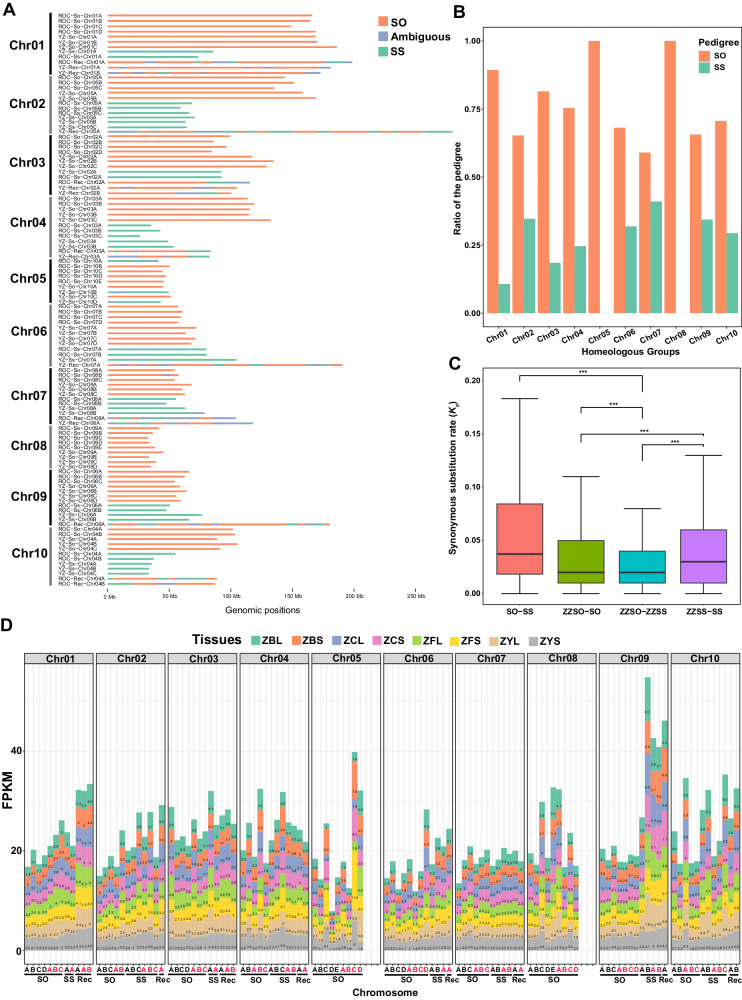
The pedigree identification and analysis of ZZ1. **A** The pedigree of *S. officinarum* (SO, colored in orange) and *S. spontaneum* (SS, colored in green) are distributed in all the allelic chromosomes of the ZZ1 genome. The ambiguous segments are colored with blue boxes. The size of each allelic chromosome was indicated by the scale at the bottom. **B** The histogram displayed the ratio of the pedigree of *S. officinarum* and *S. spontaneum* in each homologous group. The *x*-axis represents the homologous groups from Chr01 to Chr10, and the *y*-axis represents the ratio of the pedigree. **C** The divergence of the pedigree of *S. officinarum* and *S. spontaneum* in ZZ1, SO: *S. officinarum*; SS: *S. spontaneum*; ZZSO: pedigree of *S. officinarum*; ZZSS: pedigree of *S. spontaneum*; *x*-axis represents the synonymous substitution rate and *y*-axis represent the tested gene pairs from the combination of comparison. The centerline in each box represents the median; the lower and upper hinges represent the 25th and 75th percentiles, respectively; and the whiskers represent 1.5× the interquartile range. The number of gene pairs from left to right of the *x*-axis in order is as follows: *n* = 191,651, 379,051, 165,435, and 69,131. *p* values were calculated by two-sided Wilcoxon test, ^***^p < 0.001, ^**^p < 0.01, ^*^p < 0.05. **D** Total allelic expression of the chromosomes of ZZ1 in leaf and stem tested in seedling, pre-mature, and mature stages, respectively. The A–E represents the homologous chromosomes in each group, with red indicating pedigree from YZ and the black color from ROC. The pedigree of the homologous chromosomes from *S. officinarum* and *S. spontaneum*, or the recombination between them, were indicated with SO, SS, and Rec, respectively. ZBL: leaf in the pre-mature stage; ZBS: stem in the pre-mature stage; ZCL: leaf in the mature stage; ZCS: stem in the mature stage; ZFL: leaf in the tillering stage; ZFS: stem in the tillering stage; ZYL: leaf in the seedling stage; ZYS: stem in the seeding stage. Source data are provided as a Source Data file.

Using the species-specific *k*-mers extracted from the So and Ss genomes, we identified 5.4 Gb (51.9%) genomic sequences from 68 SO-originated chromosomes (ZZSO) and 1.8 Gb (17.3%) from 31 SS-originated chromosomes (ZZSS). We also detected that 1.9 Gb (18.3%) sequences in 15 chromosomes probably originated from the interspecific recombination (Rec) between the two species (Fig. 2a,b), which coincides with the results of the cytological study^19^. The differentiation in the pedigree of ZZ1 allows us to compare the transposable element (TE) content between ZZSO, ZZSS, and Rec region. We observed that, on average, 71.03% of the genome in ZZSO is comprised of TEs, which is significantly higher than the TE content in ZZSS (62.61%) and Rec (61.07%) (Supplementary Fig. 6). These findings indicate a distinct genome structure among the subgenomes, highlighting the impact of pedigree divergence on TE distribution.

The 68 SO-originated chromosomes contained different numbers of haplotypes in each homologous chromosome group, ranging from five haplotypes in Chr02/04/10 to eight in Chr06 (Fig. 2b and Supplementary Table 6). The SS-originated chromosomes had an average number of 3.1 haplotypes in the ten homologous chromosome groups, with the Chr02 group containing the most SS-originated haplotypes (i.e., six) (Fig. 2b and Supplementary Table 6). In addition, the pedigree of the *S. spontaneum* was not distributed on Chr05 and Chr08, which led to the basic chromosome number changes from 10 to 8 in *S. spontaneum*^10^. It indicates that the *S. spontaneum* with *x* = 8 was used in ZZ1 sugarcane breeding rather than the accession with *x* = 10^11^.

To investigate the divergence between the pedigrees of *S. officinarum* and *S. spontaneum* in the ZZ1 genome, we calculated the synonymous substitution (*Ks*) of the ortholog pairs of SO-ZZSO, SS-ZZSS, ZZSS-ZZSO, and SO-SS (Fig. 2c). The *Ks* analysis indicated that the ZZSO and ZZSS had the lowest divergence (Medium *Ks* = 0.020). In contrast, the SO-SS had the highest divergence (Medium *Ks* = 0.037) compared with other ortholog pairs, suggesting that recombining these two kinds of pedigrees that occurred in ZZ1 might alleviate the sequence divergence between them. The *Ks* results also exhibited that the divergence of SS-ZZSS (Medium *Ks* = 0.020) was much higher than that of SO-ZZSO (Medium *Ks* = 0.030), indicating the multiple generations of backcross with *S. officinarum* in the process of modern hybrid breeding might reduce the divergence between *S. officinarum* and its pedigrees of ZZ1. In addition, we have conducted a thorough analysis of the *Ks* values between the two subgenomes of ZZ1 and Miscanthus. The calculated *Ks* values for ZZSO-Miscanthus and ZZSS-Miscanthus are 0.0763 and 0.0764, respectively. These values indicate a similar level of divergence between the subgenomes, which aligns with the comparable divergence times observed (Supplementary Fig. 7).

### Homoeolog expression dominance in ZZ1

We collected RNA-seq data of leaf and stem in seedling, pre-mature, and mature stages to compare the expression pattern of the homoeologous chromosomes in the ZZ1 genome. Most of the chromosomes showed similar average expression levels between the SO and SS subgenomes, including Chr02, Chr03, Chr04, Chr06, and Chr07. However, the asymmetric expression patterns were detected within some homoeologous chromosome groups (Fig. 2d). For instance, it showed that the three homoeologous chromosomes derived from recombining the two foundational ancestral species were highly expressed compared with other chromosomes in the Chr01 group. The Chr09 group exhibits the dominant expression in the homoeologous chromosomes derived from SS (Fig. 2d).

**Fig. 3:**
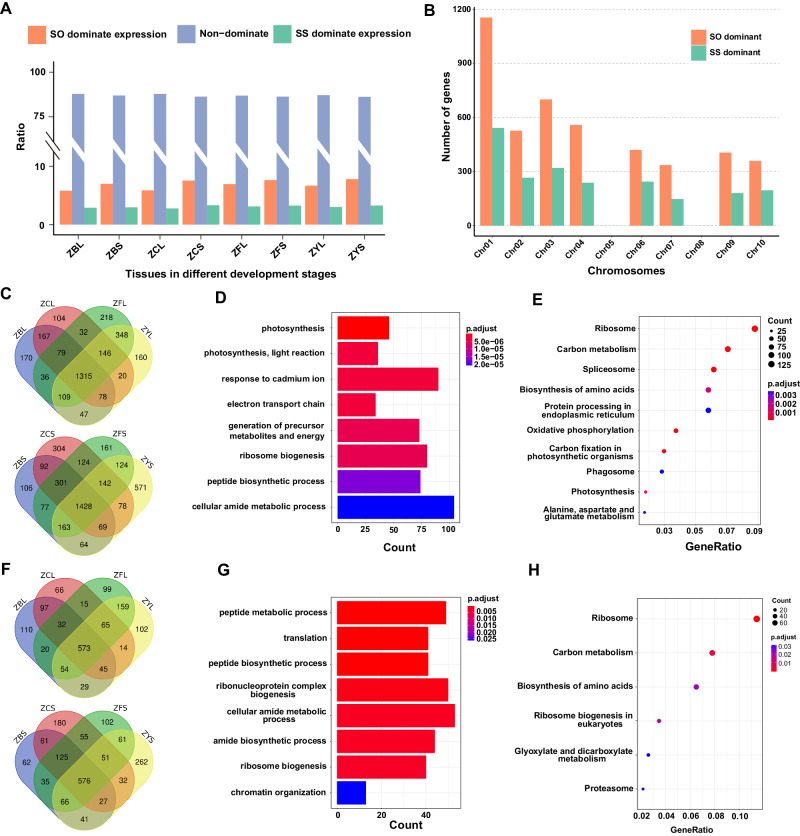
The homoeolog expression analysis in ZZ1. **A** Histograms of genome-wide expression of homoeologous genes between the pedigree of *S. officinarum* and *S. spontaneum* among ZZ1 tissues and different developmental stages. ZBL: leaf in the pre-mature stage; ZBS: stem in the pre-mature stage; ZCL: leaf in the mature stage; ZCS: stem in the mature stage; ZFL: leaf in the tillering stage; ZFS: stem in the tillering stage; ZYL: leaf in the seedling stage; ZYS: stem in the seeding stage. **B** Histograms of the distribution of dominant expressed genes in each homoeologous group. The Venn diagram of the dominant expressed genes with SO over SS (**C**) and SS over SO (**F**) in leaf and stem tissues in different development stages. The GO enrichment for the dominant expressed genes with SO over SS (**D**) and SS over SO (**G**) in all tested tissues. The KEGG enrichment for the dominant expressed genes with SO over SS (**E**) and SS over SO (**H**) in all tested tissues. The color bar represents the scale of the corrected *p* value of enrichment terms. All the significance in GO and KEGG enrichment was tested by two-tailed Fisher’s exact test method. Source data are provided as a Source Data file.

In addition, our results identified that a small proportion of homoeolog genes showed biased expression towards either *S. officinarum* pedigree (7.1% on average) or *S. spontaneum* pedigree (3.0% on average) in the RNA-seq samples examined (Fig. 3). This result indicates no significant global genome dominance between the two foundational species pedigree in ZZ1, consistent with the reported polyploid species, including *Gossypium hirsutum*^22^, *Triticum aestivum*^23^, *Brassica juncea*^24^, and *Brassica napus*^25^. GO enrichment analysis showed that those SO-dominant genes were mainly enriched in the photosynthesis-related biological processes, including “photosynthesis” and “response to cadmium ion.” However, the SS-dominant genes were primarily enriched in basic biological functions, including peptide metabolic and biosynthetic processes (Fig. 3d, g).

### The expansion of crucial gene families related to sugar transport and resistance genes

**Fig. 4:**
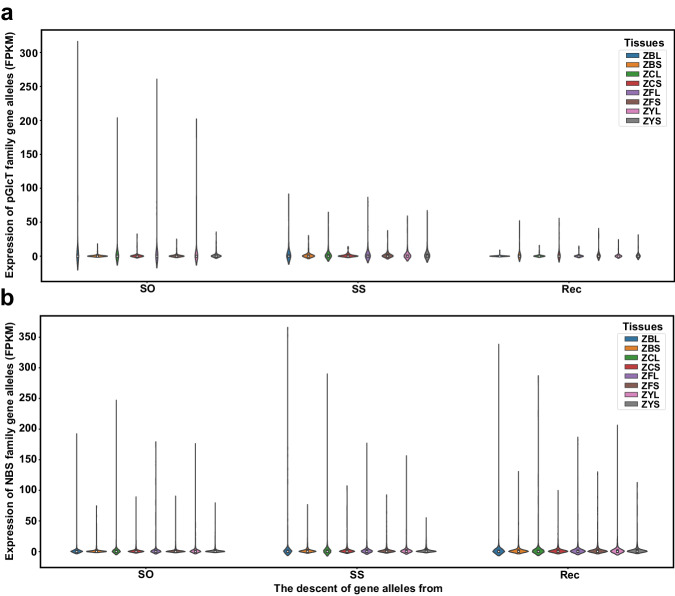
Gene family associated with the critical trait in modern sugarcane breeding. The expression of gene alleles of pGlcT (**a**) and NBS (**b**) family putative derived from SO, SS, and Rec in leaf and stem of different development stages in ZZ1 is shown by the violin plot; the *x*-axis represented the three derived lineages and *y*-axis represented the FPKM value. The legend indicates that the leaf and stem are from different stages of ZZ1 development. ZBL: pre-mature leaf; ZBS: pre-mature stem; ZCL: mature leaf; ZCS: mature stem; ZFL: the leaf in the tillering stage; ZFS: the stem in the tillering stage; ZYL: seedling leaf; ZYS: seedling stem. Source data are provided as a Source Data file.

Sugar accumulation and disease resistance are the most vital agronomic traits constantly being studied when breeding modern sugarcane cultivars. To characterize the genetic basis of these traits in the ZZ1, we analyzed the core gene families related to sugar accumulation and disease resistance (Fig. 4).

For the sugar transporter family, a total of 130 genes, including 1166 alleles, likely belong to the members of the sugar transporter superfamily consisting of polyol/monosaccharide transporters (PLT), vacuolar glucose transporter (VGT), early-responsive to dehydration protein (SFP), tonoplast monosaccharide transporters (TMT), sugar transporter protein (STP), plastidic glucose transporter (pGlcT), inositol transporter (INT), sucrose transporter (SUT), and the sugars will eventually be exported transporters (SWEET) subfamily (Table 2). Tracing of the allelic genes showed that 47.4% (SUT) to 68.2% (VGT) of alleles of these families were derived from SO, while only 7.0% (SUT) to 30.0% (INT) were from SS. In addition, we identified a substantial number of allelic genes from the reconstruction (Rec) of the two subgenomes, ranging from 13.6% of VGT to 45.6% of SUT. The expression profile of these alleles showed that the alleles derived from SO were significantly more abundant than those derived from SS across the different stages of leaf reconstruction (Fig. 4a), indicating that the genetic basis for sugar transport in ZZ1 is significantly contributed by SO. In addition, ZZ1 has more members in the PLT, TMT, and SWEET families than SS, SO, and their relative species, suggesting that gene expansion occurred in these three families during the breeding of ZZ1.

**Table 2:** The critical trait-related gene families identified in ZZ1 compared with related species

	Genes from each genome						Genes alleles of ZZ inherit from		
	Os	Zm	Sb	AP	SOL	ZZ	SO	SS	Rec
PLT	11	26	17	31	24	35	148	64	93
VGT	2	3	2	4	2	3	15	4	3
SFP	4	18	7	14	10	10	41	19	22
TMT	4	9	3	5	6	10	50	23	20
STP	21	23	22	35	29	25	143	56	53
pGlcT	1	2	1	3	2	3	16	5	4
INT	2	10	4	4	4	5	24	15	11
SUT	5	7	6	9	7	8	27	4	26
SWEET	23	23	23	22	24	31	147	64	69
NBS	476	145	311	448	665	571	2675	1267	1239

Os, rice; Zm, maize; Sb, Sorghum; AP, *S. spontaneum* AP85–441; SOL, *S. officinarum* LA-purple; ZZ, ZZ1; SO, ZZ1’s subgenome *S. officinarum*; SS, ZZ1’s subgenome *S. spontaneum*; Rec, recombination between the two subgenomes.

The nucleotide-binding site (NBS) is a vital family of plant transcription factors that regulate plant disease resistance. We identified a total of 571 putative gene members of the NBS family, which comprised 5181 alleles with 51.63, 24.45, and 23.91% of the alleles derived from SO, SS, and their Rec regions, respectively (Table 2). Although the number of NBS gene alleles derived from SS was less than that from SO, they showed higher expression than that of the alleles derived from SO (Fig. 4b), indicating that the lineage of SS might contribute to enhanced disease resistance through the crossing. We speculated that these genes might be involved in sugarcane resistance to the pathogen and a series of defense responses after infection.

### The lineages dominate expression to participate in smut and PBD

**Fig. 5:**
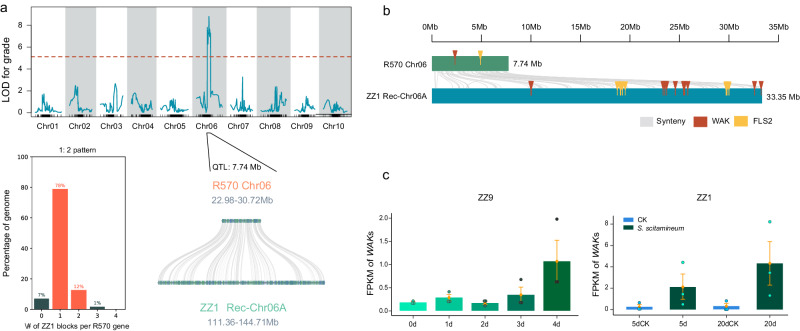
The gene family associated with resistance in modern sugarcane breeding. **a** The QTL linking to sugarcane smut was analyzed using R/QTL v1.39 software. The red dotted line indicates the filter threshold (LOD = 7.119, *p* < 0.05), and the QTL linking to sugarcane smut was obtained at the R570 Chr06 chromosome. Using the R570 QTL region (7.74 Mb) for smut resistance as a reference, QTL regions for smut resistance were identified in ZZ1, with gene collinearity shown within the QTL region connected by gray curved lines. Depicted 1:2 collinearity pattern using bar graphs. The *x*-axis represents of ZZ1 blocks per R570 gene, while the *y*-axis represents the percentage of the genome. **b** Syntenic distribution of resistance-related gene families within the homologous region. Synteny is represented by gray curved lines, the WAK gene family is indicated in deep brown, and the FLS2 gene family is represented in yellow. **c** Expression patterns of the WAK gene family. Depicted using bar graphs. The *x*-axis represents different treatment times for root and bud, while the *y*-axis represents FPKM values. Data are presented as mean values ± SE, *n* = 3. Error bars represent the standard error. Source data are provided as a Source Data file.

Smut is caused by the *Sporisorium scitamineum* (Ssc) that enters the sugarcane plant through lateral buds to colonize the apical meristem tissue. Based on gene collinearity analysis, a homologous region for smut resistance was identified on the YZ-Rec-Chr06A chromosome of ZZ1 (see Methods), spanning approximately 33.35 Mb and containing 1902 genes. Collinearity plots, region lengths, and the number of genes indicated that ZZ1 had a significant expansion compared to modern sugarcane cultivar R570^18^. Some genes exhibit a 1:2 collinearity pattern compared to R570 (Fig. 5a). The gene functional annotation reveals that in the ZZ1 genome, there is a significant presence of genes, such as flagellin sensing 2 (*FLS2*), wall-associated kinase (*WAK*), associated with plant resistance to pathogen infection, as well as genes related to cell wall formation, such as glucuronic acid xylan and hemicellulose synthesis. In this context, the WAK family in ZZ1 has increased tenfold compared to R570. The copy number of *FLS2* in ZZ1 also has increased eight-fold (Fig. 5b). The substantial amplification of these functional genes may contribute to ZZ1’s high resistance to smut, providing a genetic foundation for such resistance. The analysis of expression patterns revealed that WAK family genes exhibited an upregulated response to Ssc infection and a sustained upregulation in the growth processes of sugarcane buds, roots and leaves (Fig. 5c), indicating that WAK family genes play a crucial role in resisting pathogen infections and regulating cell proliferation and other processes.

**Fig. 6:**
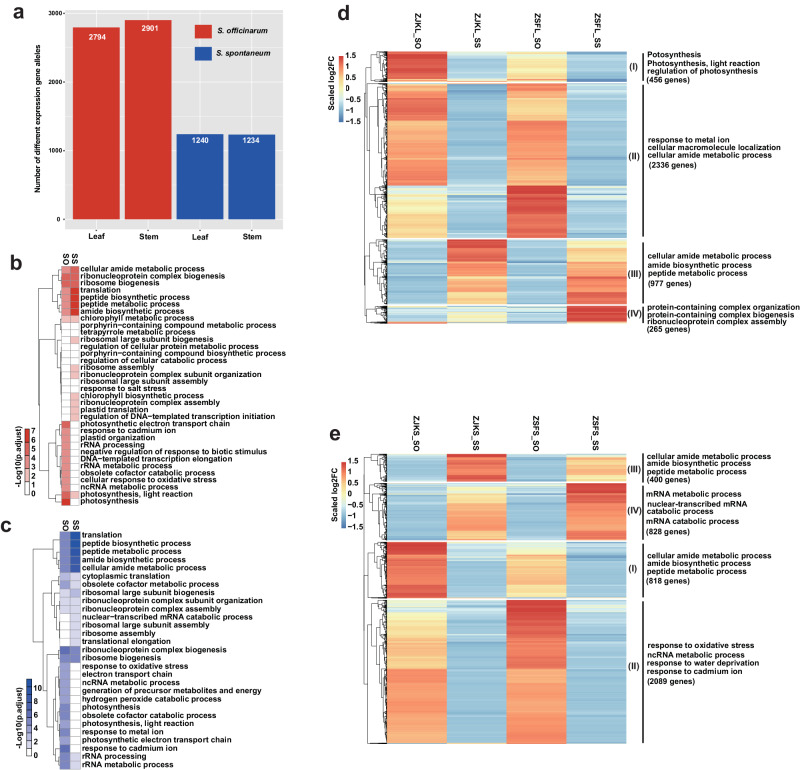
The dominant expressed genes in the SO or SS lineage responded to PBD. **a** Barplot of a total number of dominant expressed genes derived from SO or SS in response to PBD in the leaf and stem of ZZ1. **b**, **c** The heatmap of GO enrichment terms for lineage dominant expressed (LDE) genes derived from SO and SS in leaf (**b**) and stem (**c**), respectively. The color bar represents the scale of the corrected *p* value of enrichment terms. Significance was tested by the two-tailed Fisher’s exact test method. **d**, **e** Heatmap of expression changes in the SO and SS lineages between the normal growth (control) and after infection of PBD in the leaf (**d**) and stem (**e**) of ZZ1. The log10 infection/control fold-change values were normalized and scaled by *z*-score in each gene pair. The gene cluster size and representative enriched functional classification terms are noted. Source data are provided as a Source Data file.

PBD is one of the most severe diseases caused by *Fusarium fujikuroi* species Complex^26^, which resulted in catastrophic damage to the sugarcane industry. To elucidate the underlying mechanism of divergence in response to PBD among the two lineages (*S. officinarum* and *S. spontaneum*) in ZZ1, we implemented RNA-seq to survey the global transcriptomic responses to PBD in the leaf and stem tissues of ZZ1. There were 2794 and 2901 homoeologous genes from SO that showed lineage dominant expression (LDE) relative to those from SS in leaf and stem, which was greater than the number of dominantly expressed genes contributed by SS (1240 and 1234 LDE identified from leaf and stem, respectively) (Fig. 6a), suggesting the divergent response mechanism between these two lineages in ZZ1. The further GO enrichment for these genes showed that the LDE genes derived from SO lineages were mainly enriched in the photosynthesis-related process (Fig. 6b), including “photosynthetic electron transport chain,” “response to cadmium ion,” and “plastid organization.” In contrast, the ones derived from SS lineages are principally likely to participate in the process of responding to stimuli, such as “ribosomal large subunit biogenesis,” “peptide metabolic process,” “ribosome assembly,” and “tetrapyrrole metabolic process” (Fig. 6c), indicating the complex combinatorial regulation of both two lineages in response to PBD.

To unveil how the two lineages respond to PBD in ZZ1, we conducted a heatmap and clustering analysis for the LDE genes among the leaf and stem to reveal the regulation patterns in the two lineages (Fig. 6d,e). As a result, the number of LDE genes that were upregulated in SO (cluster II: 2336 genes) was greater than that in SS (cluster IV: 265 genes) in the leaves, contrary to the number of LDE genes that were downregulated in SO (cluster I: 456 genes) less than SS (cluster III: 977 genes). However, the numbers of up-and down-regulated LDE genes identified in SO [2089 up- (cluster II) and 818 down-regulated (cluster I) genes] are all more than that in SS [828 up- (cluster IV) and 400 down-regulated (cluster III) genes] in stem tissues, indicating the divergent regulation patterns in response to PBD. GO enrichment showed that the downregulated LDE genes of SO were differently enriched from that of SS in the leaf, with the former mainly enriched in photosynthesis-related terms, including “photosynthesis, light reaction”, and “regulation of photosynthesis.” In contrast, the latter is enriched in amide metabolic-related processes, such as the “cellular amide metabolic process,” “amide biosynthetic process,” and “peptide metabolic process.” The downregulated LDE genes from these two lineages are enriched in the same terms (including “cellular amide metabolic process,” “amide biosynthetic process,” and “peptide metabolic process”) in the stem tissue, suggesting the divergent regulation patterns to response to PBD occurred in these two lineages in the two tissue. Furthermore, the upregulated LDE genes from SS in the leaf and stem are mainly enriched in the vital life process, including “mRNA metabolic process,” “nuclear-transcribed mRNA,” “protein-containing complex organization,” and “ribonucleoprotein complex assembly.” The upregulated LDE genes from SO were enriched in response to the stress-related process, including “response to metal ion,” “response to oxidative stress,” “response to water deprivation,” and “response to cadmium ion,” suggesting that the LDE genes from these two lineages might play a cooperative process to respond to PBD in the leaf and stem tissues of ZZ1.

## Discussion

Despite significant advancements in the haplotype phased genome of two ancestral *Saccharum* species^10,11^, which has dramatically advanced the field of sugarcane genomics, they are still unrepresentative of the genomic information of the modern cultivated sugarcane, which possesses many advantageous traits, such as the combination of high sugar content, super abiotic stress resistance, and other exceptional traits. The resolution of these traits is reliant on the complete deciphering of high-quality modern cultivated sugarcane genomes. However, the modern cultivated sugarcane genome is one of the most intricate and challenging worldwide. Over the past two decades, sugarcane genome research pioneers have expended tremendous efforts on the genome of sugarcane cultivars, yet progress has been limited^16–18^. Unlike allopolyploids such as *B. napus*^25^, wheat^27^, and cotton^28^, modern cultivated sugarcane originated from crosses between auto-polyploid parents, followed by multiple rounds of backcrossing^2,3^. Over a long history of breeding, the bloodlines of several parents within the *Saccharum* genus have been mixed, resulting in a homo(eo)aneuploid commercially used in asexual reproduction. Approximately 10 to 20% of the chromosomes in its genome originated from recombination between the parents, and it possesses 8–14 homo(eo)logous copies of most genes. Therefore, the challenges in assembling the genome of modern cultivated sugarcane include (1) distinguishing between homozygous and heterozygous contigs and achieving chromosome-level scaffolding and (2) assembling and scaffolding chromosomes involving recombination events between the original parental lineages.

To overcome the characteristics of polyploidy and extensive recombination among ancestral subgenomes in the modern sugarcane hybrid genomes, we herein proposed an innovative ‘dimension reduction’ assembly strategy, supplemented by a variety of sequencing technologies, to decipher the modern sugarcane hybrid “ZZ1” genome completely (Fig. 1b). The quality of this ultra-complex genome, which benefited from innovations in assembly strategies and technological advances, is far superior to the previously published contig-levels SP80-3280^16^, draft chromosome-scale KK3^17^, and mosaic R570^18^ monoploid genome, which not only provides a unique perspective on the origin and evolution of modern sugarcane hybrid but also helps to analyze the molecular mechanism of excellent traits, which is of great significance for future precision breeding of sugarcane.

Distant crosses can introduce many superior genes at once, which can significantly impact the creation of new species and improve existing varieties. To create superior sugarcane germplasm, the interspecific crosse of *S. spontaneum* (with high abiotic stress tolerance) and *S. officinarum* (with high sugar content) and one or more subsequent backcrosses of *S. officinarum* have produced many of the modern sugarcane hybrids, namely ROC25 and YZ89-7. Further, the crosses of this modern sugarcane hybrid produced some of the second-generation modern sugarcane cultivars, such as ZZ1. This ingenious hybrid breeding strategy gives modern sugarcane hybrid the advantage of being derived from both ancestral parents but also makes its genome more complex due to the uneven genetic contribution of both ancestral parents from the hybridization process, non-directional loss of chromosomes during the transmission, and the new generation of Rec chromosomes from both parents. The high sequence similarity between large numbers of haplotypes in the same homologous group introduces some misjoin errors in the contig assembly process, and Rec contigs on Rec chromosomes will generate Hi-C signals with contigs of both ancestral origins, which causes great difficulties in scaffolding. Fortunately, direct biparental and original ancestral parental material availability can help to distinguish these massive disordered contigs into different groups by sequence similarity, read depth bias, and biparental ancestral unique *k*-mers strategies (see Methods). Benefiting from direct biparental and ancestral genomes, 87% of contigs were fruitfully divided into six pre-assigned groups, anchoring to 68 So chromosomes, 31 Ss, and 15 Rec chromosomes, which is consistent with karyotype analysis on the number of ZZ1 chromosomes (Fig. 1a). This multiple-grouping strategy has important implications for the resolution of other hyper-complex genomes. However, it does not exclude the fact that the availability of parents limits some hyper-complex genomic species.

The high-quality genome of ZZ1 demonstrates its chromosome origin and recombination in high resolution, expanding our knowledge of its chromosomes from the quantitative level to the specific sequence level. Previous studies have indicated the presence of three chromosome bases, namely 8, 9, and 10, in Ss^11^. However, it has remained uncertain that bases were used as parents in early sugarcane hybrid cultivars. Our genomic evidence now demonstrates that the ancestral parental chromosome base contributing to Ss consanguinity in ZZ1 is chromosome base 8, excluding bases 9 and 10. This conclusion is drawn from the observation that the Ss pedigree of the ZZ1 genome lacks intact ancestral chromosomes from Ss, specifically Chr05 and Chr08 homologous groups (Fig. 2a). This finding may also play a significant role in explaining the formation of aneuploids in hybrid sugarcane during the breeding process. The high-quality ZZ1 genome, in isolation from the FISH technique, more precisely determined the proportional genetic contribution and sequence composition of the two original ancestral parents to the modern sugarcane hybrid, which is vital for us to explore the genetic basis of superior traits in modern sugarcane hybrid.

The gene family results reconfirmed the biased contribution of Ss and So to the high abiotic stress resistance and high sugar content traits in modern sugarcane hybrid. We highlighted many genes that may play important roles, such as NBS, PLT, TMT, and the SWEET family. The total alleles of gene sequences provide the fundamental basis for studying gene-dominant expression under stress conditions. Furthermore, gene expression biases in subgenomes have been extensively observed in allopolyploids, such as *B. juncea*^24^, wheat^27^, cotton^28^, and *Nepenthes gracilis*^29^. These biases play a role in shaping the evolution of novel genes and enhancing the development of biologically vital traits. However, our findings indicated that in ZZ1, the hybrid sugarcane, no significant global genome dominance was observed between the two foundational species. This lack of dominance could be attributed, at least in part, to the fact that the sugarcane hybrid is a recently formed homo(eo)aneuploid accompanied by recombination exchange between the two foundational lineages. It is important to note that the two foundational lineages in ZZ1 exhibit significantly different numbers of alleles at the gene level. Despite this, they contribute similar total expression abundance. This intriguing phenomenon may involve epigenomic modifications and siRNA regulation, which are implicated in dosage balancing. A comprehensive analysis combining transcriptomic, epigenomic, and three-dimensional genomic tools would be beneficial to explore this exciting phenomenon further.

ZZ1, derived from ROC25 and YZ89-7, is highly resistant to smut and susceptible to PBD. Compared to the smut-susceptible modern sugarcane cultivar, ZZ1 had better physical resistance to smut, including lower stomatal density and aperture on the outermost bud scale and lower alkanol contents to reduce the germination rates of Ssc teliospores on buds. In addition, the region of smut resistance has expanded significantly, and gene expansion occurred in WAK and FLS2 families during the breeding of ZZ1. These findings provide a genetic foundation for such resistance.

## Methods

### Sample preparation and genome sequencing

The Zhong Zhe No. 1 (ZZ1) plant was cultivated in the greenhouse at Guangxi University. The young leaves from the same individual were collected for DNA extraction and genome sequencing.

Genomic DNA was extracted using a QIAGEN DNeasy Plant Mini kit (Qiagen, Hilden, Germany, catalog number 69106) and subject to library construction with an insert size of 300–500 bp. DNA quality was visually assessed using agarose gel electrophoresis (0.75%), and concentration was estimated using a spectrophotometer (Multiskan Sky Microplate 1510-01307 C, Thermo Fisher Scientific, MA, USA). DNA libraries were sequenced on the Illumina NovaSeq platform with the model of paired-end (PE) 150 bp.

To construct the high-quality SMRTbell libraries (30–50 kb), we followed the manufacturer’s protocol (https://support.10xgenomics.com/de-novo-assembly/library-prepr/doc/user-guide-chromium-genome-reagent-kit-v1-chemistry) to isolate high-molecular-weight DNA (>50 kb), which were subsequently subject to size selection by BluePippin system. A total of 224.36 Gb HiFi reads were generated on a PacBio Sequel II platform.

The young leaves from ZZ1 were collected for Hi-C library construction and sequencing. Briefly, the young leaves were fixed with formaldehyde lysed, and then the cross-linked DNA was digested using Hind III over 48 h. The sticky ends attached by biotins are proximity-ligated to form chimeric joined DNA that was physically sheared into a size of 500–700 bp and further sequenced on an Illumina NovaSeq platform.

Spore suspension of Ssc (the causative agent of smut) (1 × 10^6^ spores/mL) was inoculated at the roots and buds of ZZ9 (the same parents as ZZ1, derived from ROC25 and YZ89-7, is highly resistant to smut). Inoculated plants were placed in a constant temperature incubator at 28 °C in a medium moisturizing culture. Smut group samples were collected at 0 d, 1 d, 2 d, 3 d, and 4 d after inoculation for each of the above treatments with three duplicates. Similarly, spore suspension of Ssc was inoculated at the leaves of ZZ1. The samples were collected at 5 d and 20 d after inoculation (were collected every 5 d), with water as a control treatment. In addition, the different tissues and different developmental stages were collected from ZZ1, including “leaf in pre-mature stage (ZBL), stem in pre-mature stage (ZBS), leaf in mature stage (ZCL), stem in mature stage (ZCS), leaf in tillering stage (ZFL), stem in tillering stage (ZFS), leaf in seedling stage (ZYL), and stem in seeding stage (ZYS).” Total RNA was extracted from the above samples using RNAprep Pure plant Kit (Tiangen Biotech, Beijing, China, catalog numbers DP432) and subsequently used for cDNA library construction. The quality of the cDNA library was assessed on the Agilent Bioanalyzer 2100 system and sequenced on an Illumina Novaseq platform. The original data was quality controlled by Cutadapt, and the quality control data was compared to the ZZ1 genome using HISAT2 v2.1.0 software^30^. Using Cufflinks software, the expression levels of transcripts and genes were quantified through the position information of Mapped Reads on the genome. FPKM (fragments per kilobase of exon per million fragments mapped) was used as an index to measure the expression level of transcripts.

### Contig assembly

We first used Hifiasm^31^ to assemble PacBio HiFi reads with default parameters. The resulting contigs contain a total of 11.2 Gb sequences, which is much larger than the estimated genome size. It could be caused by assembly errors and contaminated sequences, introducing artifactual sequences that are not supposed to be present in the assembly. To provide a high-quality genome assembly, we identified the misjoined contigs based on abnormal Hi-C signals. The Hi-C reads aligned against the genome assembly using BWA-MEM algorithm^32^ with ‘-SP5M’ parameters, allowing split alignments suitable for Hi-C reads mapping. We further constructed the chromatin contact map within contigs and identified chimeric errors if they show substantial differences between two adjacent bins in the Hi-C signal matrix, which follows the same correction algorithm in 3D-DNA^33^ with re-compilation using Python for acceleration (https://github.com/tangerzhang/ALLHiC/blob/master/bin/ALLHiC_pip.sh). The artifactual sequences were detected and removed if they contained a large proportion (>40%) of *k*-mers present only in assembly but absent in the sequencing reads implemented in the Merqury program^34^. To identify the contaminated sequences derived from organelles and bacteria, the plant chloroplast and mitochondria genomes, along with bacterial genomes downloaded from NCBI (accessed on Oct. 2021), were aligned against the ZZ1 genome assembly using BLASTN program with an *e*-value of 10^−5^. Contigs that have more than >40% genomic regions overlapped with contaminated sequences were removed from the assemblies. The process of quality control resulted in a total of 10.4 Gb high-quality genome sequences in our assembly.

### Chromosomal-level genome assembly

The basic idea to accomplish this ultra-complex genome is to reduce the scaffolding complexity through the pre-definition of homologous groups, followed by separating different haplotypes (Fig. 1). We first assigned these assembled contigs into two groups (ROC and YZ) representing sequences from two parental genomes. This step can be achieved by two strategies based on read depth and parental-specific *k*-mers. For the read depth-based strategy, we aligned the parental short reads (ROC25 and YZ89-7) against these assembled contigs using BWA-MEM algorithm^12^ with default parameters, respectively. The normalized read depth across all the contigs was calculated using our previously developed CNV caller (https://github.com/sc-zhang/popCNV). Contigs with a 1.5× fold-change of depth biased toward ROC25 were assigned to the ROC-derived group and vice versa. In addition, the parental-specific *k*-mers were identified by comparing 21-mers between ROC25 and YZ89-7 genomic sequences. The parental-specific *k*-mers were traced back to these assembled contigs, which were subsequently assigned to the ROC or YZ groups based on a twofold change of parental-specific 21-mers.

The ancestor-derived contigs were identified based on genome assemblies and population resequencing of the two fundamental ancestral species, *S. officinarum* and *S. spontaneum*. We randomly selected five resequenced *S. officinarum* and five resequenced *S. spontaneum* individuals from our previously published data and mapped these reads against the assembled contigs^11^. Following the aforementioned procedure, the read depth for each contig was calculated and normalized using popCNV. We assigned these contigs to the SO-derived group if they showed uniformly and significantly higher read depth in *S. officinarum* resequenced samples than in *S. spontaneum* with the cutoff *p* value of 0.05 at the student’s *t*-test and vice versa. For those unassigned contigs that did not show a significant difference in read depth between the two ancestral species, we aligned these contigs against the previously published *S. spontaneum* AP85–441^10^ and *S. officinarum* LA-purple^11^ genomes using minimap2^35^ and determined the origination for each contig based on the similarity with the two ancestral genomes. Contigs with higher mapping quality and similarity with the *S. spontaneum* AP85–441 genome were re-assigned to the SS-derived group and vice versa (SO-derived).

The basic idea that Hi-C technology can be used for chromosome-scaffolding is based on the observation that intra-chromosome sequences are highly interacted in comparison with inter-chromosome contigs. It suggests that the contigs involved in recombination between SO and SS subgenomes share a high Hi-C signal density. Following this idea, we mapped previously published Hi-C reads against assemblies in *S. spontaneum* AP85-441^10^ and *S. officinarum* LA-purple^11^ genomes using BWA-MEM algorithm^12^ with ‘-SP5M’ parameters, respectively, and separated contigs into two groups SO- and SS-derived. We calculated and normalized the Hi-C signals between the SO- and SS-derived contigs with the following formula **1**:

1 $${{{{{\rm{HiC}}}}}}\; {{{{{\rm{signal}}}}}}=\frac{{{{{{\rm{HiC}}}}}}\; {{{{{\rm{link}}}}}}\; {{{{{\rm{count}}}}}}\times 2}{{{{{{\rm{contig}}}}}} 1 \, {{{{{\rm{length}}}}}}+{{{{{\rm{contig}}}}}} 2 {{{{{\rm{length}}}}}}} \times \frac{1}{{{{{{\rm{seq}}}}}}\; {{{{{\rm{depth}}}}}}}$$

This analysis identified the normalized Hi-C signal density of 0.4 as the threshold that can confidently distinguish whether the contigs are from recombined or non-recombined chromosomes (Supplementary Fig. 8). Based on the threshold, we got 1.9 Gb sequences from different ancestors that highly interacted with 0.78 Gb from ROC and 1.12 Gb from YZ. These contigs were considered sequences from the recombined chromosomes and thereafter assigned to ROC-Rec and YZ-Rec groups.

The aforementioned steps classified the assembled contigs into six groups: ROC-SO, ROC-SS, ROC-Rec, YZ-SO, YZ-SS, and YZ-Rec. Contigs within each group were subject to the ALLHiC haplotype-phasing pipeline independently, following the detailed description to assemble the auto-tetraploid sugarcane genome in Github (https://github.com/tangerzhang/ALLHiC/wiki/ALLHiC:-scaffolding-an-auto-polyploid-sugarcane-genome). The resulting scaffolds were manually inspected and adjusted based on two pieces of evidence, chromatin interaction heatmap that was implemented in juicebox and synteny relationship with the monoploid assemblies of two ancestral genomes revealed by our developed tool CATG (Collinearity-based Assembly correcTor GUI). The CATG program is a GUI application that manually adjusts genome assembly according to the collinearity with a reference genome. The codes and a user-friendly manual are openly accessible (https://gitee.com/tanger-lab_enterprise/CATG).

The quality of genome assembly was assessed using a series of approaches. Initially, the completeness of genome assembly was evaluated based on 1614 benchmarking universal single-copy orthologs collected from the Embryophyta_odb10 database^36^. We also applied a *k*-mer-based strategy to investigate the assembly consensus (i.e., quality value or QV) and completeness of these genomes, which was implemented in the Merqury program^34^. The copy number spectrum plots show that single-copy *k*-mers are dominant across these genomes with a high level of *k*-mer completeness (95.99). The QV in the genome assembly is 50.27, corresponding to more than 99.99% single-base accuracy. The genome consistency was assessed by aligning the Illumina short reads, which shows that almost all the genomic regions can be covered by more than 99.9% of sequencing reads. The chromosomal-scale genome assembly was assessed using a chromatin contact map and synteny with the sorghum genome^21^.

### Genome annotation

Repeated sequences were annotated in collaboration with RepeatModeler (http://www.repeatmasker.org/RepeatModeler/) and RepeatMasker^37^. Briefly, RepeatModeler searches for repeated sequences and generates a library using the RECON and RepeatScout algorithms^38^. Customized for the task, this library of repetitive sequences contains a variety of consensus sequences, most of which belong to the TE family. This library is subsequently utilized as the entry data for the RepeatMasker.

The protein-encoding gene annotation of the ZZ1 genome was undertaken following the pipeline illustrated by GETA. The pipeline invoked various programs such as HiSAT2^30^, augustus^39^, trimmomatic^40^, and genewise^41^ to provide evidence of homologous proteins and transcripts to support the results of the gene ab initio predictions. During the first step, we covered the ZZ1 assemblies using repetitive sequences, which could efficiently diminish the background noise generated by replicated sequences. HiSAT2 is then used to align the trimmomatic quality-controlled RNA-seq data to the assembly results. For reliable intron sequences and to validate the transcript data, GETA was performed to compute a threshold for the sequencing depth of each alignment region and filter out any transcripts below this threshold for depth. Residual high-quality transcripts were utilized by the TransDecoder software as predictive data for the ORF (https://github.com/TransDecoder/TransDecoder). Using Augustus software, a Hidden Markov Model was trained on the results based on the exon and intron predictions. Orthologous protein sequences, including those of *Arabidopsis thaliana*, *Oryza sativa*, and *Sorghum bicolor*, were gathered and presented in genewise software alongside those of the two ZZ1 ancestral parental genomes, represented by LA-Purple and AP85-441. We jointly obtained high-quality gene models derived from conserved homologous sequences, expression, and Augustus supporting scores. A homemade Perl pipeline, GetaFilter, was next employed to screen the predicted genes. To summarize, we used the Pfam database and the plant UniProt database to tailor the predictions and yield highly conserved protein sequences. Moreover, we measured the FPKM of RNA-seq data acquired from multiple tissues of ZZ1 to predict. We retained genes that met at least one of the following conditions: first, the presence of a structural domain in the Pfam database or a homologous protein in the plant UniProt database; second, FPKM above three and paired coverage above 80%; and third, Augustus support above 80.

The functional annotation of the protein-coding genes was performed based on the alignment of those deduced proteins against several databases, namely Gene Ontology (GO)^42^, Kyoto Encyclopedia of Genes and Genomes (KEGG)^43^, auxiliary proteins (TrEMBL), protein sequences (Swiss-Prot)^44^ and Clusters of Orthologous Groups (KOG)^45^.

To distinguish homologous genes between subgenomes and accurately identify alleles among homologous chromosomes, we developed the following pipeline, which includes the three major steps:

Identification of monoploid genomes and representative genes from the two ancestral genomes. Our previous study generated the haplotype-resolved assemblies for the two ancestral genomes, *S. spontaneum*^10^ (Ss, 2*n* = 4*x* = 32) and *S. officinarum*^11^ (So, 2*n* = 8*x* = 80) genomes. Using the two fully phased genome assemblies, we first generated the monoploid genomes containing only one set of haplotypes by identifying and removing redundant sequences (i.e., allelic sequences) with high similarities, which was implemented in our previously developed Khaper program^46^. This analysis resulted in an 802-Mb monoploid genome for Ss and a 1.15-Gb monoploid genome for So. We further annotated the two monoploid genomes, leading to 34,010 representative protein-coding genes in Ss and 39,355 in So.

Detection of allelic genes in the ZZ1 genome based on ancestral monoploid genomes. To separate homologous genes originating from different subgenomes, we used the monoploid genomes of the two ancestors, with a total of 73,365 protein sequences, as reference. The predicted ZZ1 proteins were BLASTed against the reference sequences, and the best match were retained using the parameters “-evalue 1e-5 -best_hit_score_edge 0.05 -best_hit_overhang 0.25 -max_target_seqs 1”. A total of 97.20% (359,751/370,103) of the ZZ1 predicted protein sequences were assigned to any of those 52,866 ancestral genes. We further identified the allelic genes based on three strategies: protein similarity, synteny, and coordinates, following a similar approach^10^. Allelic genes were partitioned into the same allelic group if they were located in the same synteny blocks identified by MCScanX^47^ and shared a high level of identity (≥70%) and coverage (≥60%). In addition, we also used the coordinating approach to determine allelic genes within the same loci among different haplotypes. The coding sequences of candidate alleles were aligned onto ZZ1 genomes, limiting the number of matches to the maximum number of the ploidy on the reference genome. This process was performed by minimap2^35^ with parameters “-x splice -k 12 -a -N 12”. Subsequently, genes with more than 50% overlap within the same loci were considered candidate allelic genes. A total of 236,845 high-quality allelic genes were retained in the initial allelic table, which contains 45,209 allele-defined genes, with 20,665 in the Ss subgenome and 24,544 in the So subgenome. We further refined the allelic table by assigning un-anchor genes based on the second round of protein alignment. This led to 69,680 genes with defined alleles in the final allelic table.

Identification of homoeologous genes between the two subgenomes. We first used blastn^48^ to search for optimal matches between So and Ss monoploid cds sequences. The parameter is “-evalue 1e-5-best_hit_score_edge 0.05-best_hit_overhang 0.25-max_target_seqs 1”. A total of 18,525 reciprocal blast hits (RBH), orthologs between Ss and So genomes, were identified. According to the correspondence of orthologs between two ancestral genomes, 10,254 genes were annotated with homoeologous genes between two subgenomes of the “ZZ1” genome in the alleles table, which was obtained in the previous step.

### Homoeolog expression dominance analysis

The clean reads from RNA-seq after qualifying control by trimmomatic (v0.38) were mapped against the homoeologous gene pairs using bowtie2^49^. The expression level of each homoeologous gene was calculated by align_and_estimate_abundance.pl of Trinity package^50^. The differentially expressed homoeologous gene pairs with greater than a twofold change were defined as dominant expression gene pairs. The dominant expression genes were relatively higher expressed in the homoeologous pairs, while the lower ones were the subordinate genes. The homoeologous gene pairs that showed non-dominance were defined as neutral genes.

### Analysis of synonymous substitution rates (Ks)

Paralogous and orthologous gene pairs were identified using the MCScanX software (http://chibba.pgml.uga.edu/mcscan2/) based on the syntenic blocks with the defeat parameter. The number of synonymous substitutions per synonymous site (*Ks*) of each gene pair was calculated using the Nei-Gojobori method (https://github.com/tanghaibao/bio-pipeline/blob/master/synonymous_calculation/synonymous_calc.py).

### Identification of homologous QTL for smut-resistance

We screened the large F1 population (c. 17,000 individual clones) of parental (ROC25 × YZ89-7) and constructed a smut-resistant/susceptible subpopulation. After field evaluation for three consecutive years, a total of 401 clones were verified, and each clone was assigned a resistance value (from 1 to 8, 1 representing the most resistant and 8 representing the most susceptible). Genotyping by Sequencing (GBS) technology was employed to sequence ROC25, YZ89-7, and 236 clones from the F1 population. This approach yielded 12,975,602 SNP sites, from which 1196 bin markers were derived after screening and filtering. Utilizing R570^18^ as the reference genome, these bin markers were utilized to construct a high-density genetic map of sugarcane, spanning a total length of 701.855 cM, with individual linkage groups varying from 59.855 cM to 81.681 cM. Subsequently, simplified genome sequencing was conducted on 220 individuals exhibiting diverse resistance levels in field conditions. Based on constructing the high-density genetic map for sugarcane, permutation calculation used disease grade as phenotypic data and determined the screening threshold to be 7.119. Next, QTL positioning analysis was conducted using R/QTL v1.39 software, employing Composite Interval Mapping (CIM) to depict a curve graph based on the LOD value of each site. Results indicated the initial location of the smut QTL on the Chr06 chromosome of the R570 genome. The QTL confidence interval, spanning 5 cM from both ends of the peak and declining by 1.5 LOD values (Fig. 5a), revealed a phenotypic variance explanation of 22.74%. The confidence interval length was 2.967 cM, spanning ~7.74 Mb, and containing 512 genes within the QTL interval.

As a reference, the ZZ1 genome was used as queries. The JCVI (python version MCScan) was employed to locate the chromosomes in ZZ1, each having the maximum number of collinear genes with the QTL region for smut resistance. Subsequently, the identified chromosomes were used as a query. JCVI was utilized to locate the regions with the most densely populated collinear genes as the QTL regions for smut resistance in R570 on homologous chromosomes of ZZ1, as well as used to construct collinearity maps between ZZ1 and the smut QTL regions of R570, and eggNOG-mapper was employed to functionally annotate genes within the QTL regions for smut resistance in all two modern sugarcane cultivar varieties.

### Gene family identification

The Hidden Markov Model (HMM) associated with conserved structural domains of each tested gene family was found in the Pfam database. All the gene family members were searched in ZZ1, *S. spontaneum* AP85-44^10^, *S. officinarum* LA-Purple^11^, Sorghum^21^, Rice^51^, and maize^52^ genome, respectively, using HMMER software with *E*-value <1E-5 base on the HMM model. All the identified gene family members were further blasted into the NCBI database for manual checking.

### Reporting summary

Further information on research design is available in the Nature Portfolio Reporting Summary linked to this article.

### Supplementary information

Supplementary Information

Peer Review File

Description of Additional Supplementary Files

Supplementary Data 1

Reporting Summary

### Source data

Source Data

## Data Availability

The raw sequence reads, genome assembly, and annotation data generated in this study have been deposited in the China National Center for Bioinformation Genome Warehouse under accession code GWHEQVP00000000 [https://ngdc.cncb.ac.cn/gwh/Assembly/83532/show]. The genome data of *S. officinarum* LA-Purple are available at NCBI under Bioproject accession PRJNA744175. The genome data of KK3 were downloaded from NCBI under accession JALQSO000000000. The genome data of SP80-3280 were downloaded from NCBI under accession GCA_009173535.1. The genome data of R570 were downloaded from the sugarcane genome hub [https://sugarcane-genome.cirad.fr/content/download]. The genome data of AP85–441 were downloaded from NCBI under accession QVOL00000000 [https://www.ncbi.nlm.nih.gov/datasets/genome/GCA_003544955.1/]. The genome data of Np-X were available at the Sequence Read Archive under BioProject accession PRJNA721787. The genome data of rice are available at the National Genomics Data Center under PRJCA005549. The genome data of sorghum were downloaded from NCBI under accession ABXC00000000.3. The genome data of maize were downloaded from NCBI under accession LPUQ00000000. RNA-Seq raw data have been deposited in NCBI under accession PRJNA1083323. The public databases used in this study include UniProt database (http://www.uniprot.org/), GO database (http://geneontology.org/), KEGG database (https://www.kegg.jp), Swiss-Prot database (http://www.uniprot.org/downloads), TrEMBL (http://www.expasy.org/sprot), KOG database (ftp://ftp.ncbi.nih.gov/pub/COG/KOG/kyva), and Pfam database (http://ftp.ebi.ac.uk/pub/databases/Pfam/current_release). Source data are provided in this paper. Source data are provided with this paper.

## Code availability

The Khaper algorithm is available at GitHub [https://github.com/lardo/khaper]. ALLHiC can also be found at GitHub [https://github.com/tangerzhang/ALLHiC/wiki/ALLHiC:-scaffolding-an-auto-polyploid-sugarcane-genome]. Codes were also archived on Zenodo [10.5281/zenodo.4780792].
