# Supplementary material for: A chromosomal-scale genome assembly of modern cultivated hybrid sugarcane provides insights into origination and evolution
Source: Nat Commun. 2024 Apr 8;15:3041. doi: 10.1038/s41467-024-47390-6 (PMC11001919; doi:10.1038/s41467-024-47390-6)
Supplement: Supplementary file 1 — Supplementary Information [file 41467_2024_47390_MOESM1_ESM.pdf]

Supplementary Table 1. Correspondence table of sugarcane chromosome nomenclature.

<table>
<tr><th rowspan="2">Sorghum</th><th rowspan="2">S. spontaneum Np-X</th><th rowspan="2">S. spontaneum AP85-441</th><th colspan="3">R570</th><th colspan="3">ZZ1</th></tr>
<tr><th>STP</th><th>SS</th><th>SO</th><th>Chr. groups</th><th>SS</th><th>SO</th></tr>
<tr><td>Sb01</td><td>Chr01</td><td>Chr1</td><td>Sh01</td><td>1</td><td>1</td><td>Chr01</td><td>Ss-Chr01</td><td>So-Chr01</td></tr>
<tr><td>Sb02</td><td>Chr02</td><td rowspan="2">Chr2</td><td>Sh02</td><td rowspan="2">5</td><td>5</td><td>Chr02</td><td rowspan="2">Ss-Chr05</td><td>So-Chr05</td></tr>
<tr><td rowspan="2">Sb08</td><td rowspan="2">Chr08</td><td rowspan="2">Sh08</td><td rowspan="2">9</td><td rowspan="2">Chr08</td><td rowspan="2">So-Chr09</td></tr>
<tr><td rowspan="2">Chr7</td><td rowspan="2">6</td><td rowspan="2">Ss-Chr06</td></tr>
<tr><td>Sb09</td><td>Chr09</td><td>Sh09</td><td>6</td><td>Chr09</td><td>So-Chr06</td></tr>
<tr><td>Sb06</td><td>Chr06</td><td rowspan="2">Chr5</td><td>Sh06</td><td rowspan="2">7</td><td>8</td><td>Chr06</td><td rowspan="2">Ss-Chr07</td><td>So-Chr07</td></tr>
<tr><td rowspan="2">Sb05</td><td rowspan="2">Chr05</td><td rowspan="2">Sh05</td><td rowspan="2">10</td><td rowspan="2">Chr05</td><td rowspan="2">So-Chr10</td></tr>
<tr><td rowspan="2">Chr6</td><td rowspan="2">8</td><td rowspan="2">Ss-Chr08</td></tr>
<tr><td>Sb07</td><td>Chr07</td><td>Sh07</td><td>8</td><td>Chr07</td><td>So-Chr08</td></tr>
<tr><td>Sb03</td><td>Chr03</td><td>Chr3</td><td>Sh03</td><td>2</td><td>2</td><td>Chr03</td><td>Ss-Chr02</td><td>So-Chr02</td></tr>
<tr><td>Sb04</td><td>Chr04</td><td>Chr4</td><td>Sh04</td><td>3</td><td>3</td><td>Chr04</td><td>Ss-Chr03</td><td>So-Chr03</td></tr>
<tr><td>Sb10</td><td>Chr10</td><td>Chr10</td><td>Sh10</td><td>4</td><td>4</td><td>Chr10</td><td>Ss-Chr04</td><td>So-Chr04</td></tr>
</table>

Supplementary Table 2. Statistics of genome sequencing.

	Parental Illumina sequencing		Sequencing statistics of ZZ1 clean reads		
	**ROC25**	**YZ89-7**	**Illumina**	**PacBio HiFi**	**Hi-C**
Data size（Gb）	528.48	555.75	656.47	224.36	1175.38
Max length (bp)	150	150	150	38,093	150
Reads N50 (bp)	150	150	150	13,219	150
Average length (bp)	150	150	150	13,145	150
Number of sequences	1,438,179,444	1,512,375,548	1,786,473,754	20,536,458	3,209,617,098

Supplementary Table 3. Statistics of Hi-C mapping.

**Statistics of mapping**	
Clean Paired-end Reads	1,093,551,318
Unmapped Paired-end Reads	40,376,761
Unmapped Paired-end Reads Rate (%)	3.69
Paired-end Reads with Singleton	380,762,878
Paired-end Reads with Singleton Rate(%)	34.819
Multi Mapped Paired-end Reads	558,194,001
Multi Mapped Ratio (%)	51.04
Unique Mapped Paired-end Reads	114,217,678
Unique Mapped Ratio (%)	10.45
**Statistics of valid reads**	
Unique Mapped Paired-end Reads	114,217,678
Dangling End Paired-end Reads	21,726,183
Dangling End Rate (%)	19.02
Self Circle Paired-end Reads	485,026
Self Circle Rate (%)	0.42
Dumped Paired-end Reads	133,386
Dumped Rate (%)	0.12
Interaction Paired-end Reads	77,138,022
Interaction Rate (%)	67.54
Lib Valid Paired-end Reads	59,906,451
Lib Valid Rate (%)	77.66
Lib Dup (%)	22.34

Supplementary Table 4. Statistics of contig-level assembly.

	**Hifiasm unitigs**	**Hi-C corrected and clean unitigs**
No. of contigs	141,261	186,606
Max length (Mb)	19.7	16.2
Assembly size (Gb)	11.2	10.4
Contig N90 (bp)	26,287	24,840
Contig N50 (bp)	527,183	125,000
Average (bp)	79,430	55,571

Suppplementary Table 5. Assignment of contigs into parents and ancestral parents.

		No. of contigs	Size (Mb)	Percentage of assembled size (%)
Parental Group	Roc	61,582	4,321.67	41.67
	YZ	62,466	4,724,74	45.56
	Unassigned	62,558	1,323.61	12.76
	Total	186,606	10,370.02	100
Ancestral Group	So	95,642	5,341.70	51.51
	Ss	11,721	1,788.69	17.35
	Rec	16,685	1,905.02	18.37
	Unassigned	62,558	1,323.61	12.76
	Total	186,606	10,370.02	100

Supplementary Table 6. Statistics of haplotype-resolved, chromosome-scale assembly.

	ROC					YZ			
	**A**	**B**	**C**	**D**	**E**	**A**	**B**	**C**	**D**
SoChr01	154.58	151.56	137.84	155.15		153.55	153.48	170.30	
SoChr05	134.12	141.49	125.86			143.83	156.20		
SoChr02	94.02	80.82	88.92	77.80		106.68	124.03	118.94	
SoChr03	104.25	108.01				106.18	103.39	121.33	
SoChr10	39.53	45.81	41.32	44.80	43.43	41.95	46.73	46.71	40.54
SoChr07	53.85	55.79	56.16	53.38		66.26	57.89	65.22	63.17
SoChr08	49.46	52.45	49.38			62.01	55.03	58.57	
SoChr09	39.83	32.62	30.15	31.29	34.99	41.23	30.33	36.79	32.52
SoChr06	62.28	59.24	50.44			55.10	60.10	50.77	54.08
SoChr04	91.97	94.88				81.01	97.13	82.75	
SsChr01	69.93					80.95			
SsChr05	64.10	56.90	63.17			66.85	60.29	61.09	
SsChr02	88.31					89.29			
SsChr03	34.10	42.23	25.54			47.59	52.63		
SsChr07	77.40	76.90				102.92			
SsChr08	51.72	42.87				59.13	71.15		
SsChr06	48.62	45.10				73.93	62.43		
SsChr04	53.46	35.91				33.70	30.91	31.74	
RecChr01	180.46					162.54	150.71		
RecChr05						261.04			
RecChr02	104.70					97.61	90.79		
RecChr03	77.25					75.78			
RecChr07						172.85			
RecChr08	95.98					111.26			
RecChr06	166.28								
RecChr04	81.11	78.32							

Supplementary Table 7. Comparison of assembly parameters of ZZ1 with previously published sugarcane genomes.

	**Sugarcane cultivars (homo(eo)aneuploid)**				**Sugarcane wild species**	
	ZZ1	R570[1]	KK3[2]	SP80-3280[3]	*S. spontaneum* Np-X[4]	*S. spontaneum* AP85-441[5]
**Total of scaffolding Chromosomes**	114	10	56	-	40	32
**size of assembly (Gb)**	10.400	0.382	7.0	4.260	2.760	3.130
**Contig N50 (Kb)**	125	-	83	13	381	45
**No. of annotated gene (alleles)**	257,534	25,316	242,406	373,869	122,441	112,788
**BUSCO (%)**	99.7	-	-	90.9	96.46	97.01

Supplementary Table 8. BUSCO assessement of genome assembly.

Derscription	Hi-C corrected unitigs	
	Number	Percentage (%)
Complete BUSCOs(C)	1609	99.7
Complete and single-copy BUSCOs(S)	20	1.2
Complete and duplicated BUSCOs(D)	1589	98.5
Fragmented BUSCOs(F)	1	0.1
Missing BUSCOs(M)	4	0.2
Total BUSCO groups searched	1614	100

Supplementary Table 9. Assessment of genome consistency based on Illumina reads.

Item	Statistics
Number of clean reads*	3,247,365,242
Data size (Gb)	487.10
Mapped bases (Gb)	486.81
Mapping rate (%)	99.94
Genome length (Mbp)	10,382.41
Mean depth	46.92
Coverage rate (%)	100.00
Regions with low coverage (< 5 reads)	0.00
Percentage with low coverage (< 5 reads; %)	0.00

* Contaminated and low-quaility reads were removed from the FASTQ files.

Supplementary Table 10. Annotation of transposable elements in the cultivated sugarcane genome.

	Total		Rec		So		Ss	
	**Length (Mb)**	**% of genome**	**Length (Mb)**	**% of genome**	**Length (Mb)**	**% of genome**	**Length (Mb)**	**% of genome**
Total repeat fraction	6907.99	66.54	1158.03	11.15	3785.15	36.45	1126.29	10.84
Class I: Retroelement	4737.08	45.63	775.04	7.45	2655.57	25.56	759.62	7.3
LTR Retrotransposon	4496.94	43.31	724.42	6.97	2537.63	24.44	719.37	6.92
Ty1/Copia	1625.25	15.65	301.57	2.9	920.45	8.86	228.26	2.19
Ty3/Gypsy	2847.21	27.42	418.64	4.03	1605.30	15.46	487.11	4.69
Other	24.48	0.24	4.21	0.04	11.88	0.12	4.00	0.04
Non-LTR Retrotransposon	240.15	2.31	50.61	0.48	117.94	1.12	40.25	0.38
LINE	218.61	2.11	46.06	0.44	107.66	1.03	36.42	0.35
SINE	21.54	0.21	4.55	0.04	10.29	0.09	3.83	0.03
Class II: DNA Transposon	936.20	9.02	188.12	1.81	454.03	4.37	163.51	1.57
TIR								
CMC[DTC]	337.34	3.25	57.62	0.55	162.93	1.56	63.55	0.61
hAT	99.89	0.96	20.38	0.19	50.07	0.48	16.66	0.16
MULE-MuDR	107.61	1.04	23.28	0.22	51.71	0.49	18.53	0.17
PIF/Harbinger	255.14	2.46	56.32	0.54	126.13	1.21	39.80	0.38
Helitron	48.28	0.46	9.93	0.09	22.52	0.21	9.43	0.09
Simple Repeats	75.63	0.73	15.35	0.14	35.16	0.33	12.51	0.12
Unknown	866.54	8.35	140.51	1.35	498.06	4.79	123.00	1.18

Supplementary Table 11. Annotation of homoelogous gene pairs between two sub-genomes in ZZ1.

	So-Ss homoeologous gene pairs	So-duplicated genes	So-specific genes	Ss-duplicated genes	Ss-specific genes	Unassigned genes
Chr01	5,270	10,585	2,879	7,609	399	10,955
Chr02	3,091	6,758	1,559	12,918	623	5,342
Chr03	3,304	7,379	1,782	5,241	396	4,978
Chr04	2,379	6,164	1,239	5,554	356	2,605
Chr05	1,495	1,878	851	NA	NA	2,236
Chr06	2,038	5,470	1,131	5,509	468	4,224
Chr07	1,362	3,351	858	3,917	419	4,046
Chr08	915	1,501	586	NA	NA	1,242
Chr09	1,696	4,388	847	4,058	508	3,007
Chr10	2,120	4,062	1,037	3,369	411	3,035
Total	23,670	51,536	12,769	48,175	3,580	41,670

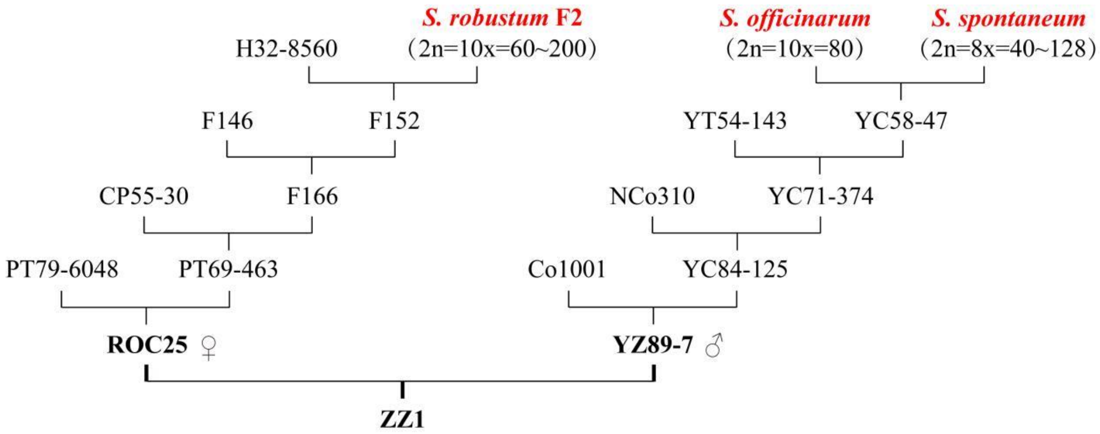

Supplementary Figure 1. The breeding history of the modern hybrid sugarcane ZZ1.

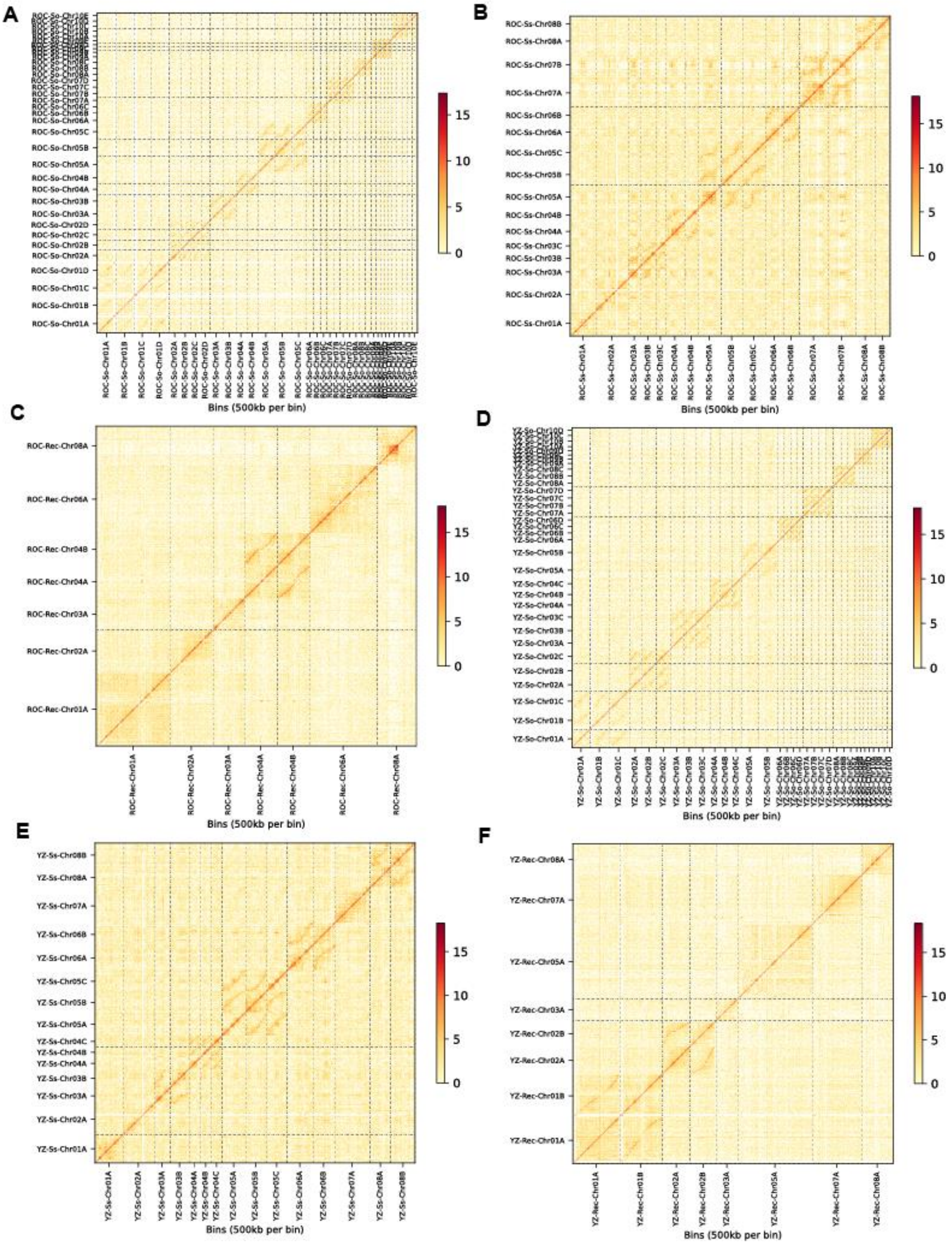

Supplementary Figure 2. Genome-wide analysis of chromatin interactions at 500-kb resolution in six pre-assigned groups among ZZ1 genome. A. ROC-So; B. ROC-Ss; C. ROC-Rec; D. YZ-So; E. YZ-Ss; F. YZ-Rec.

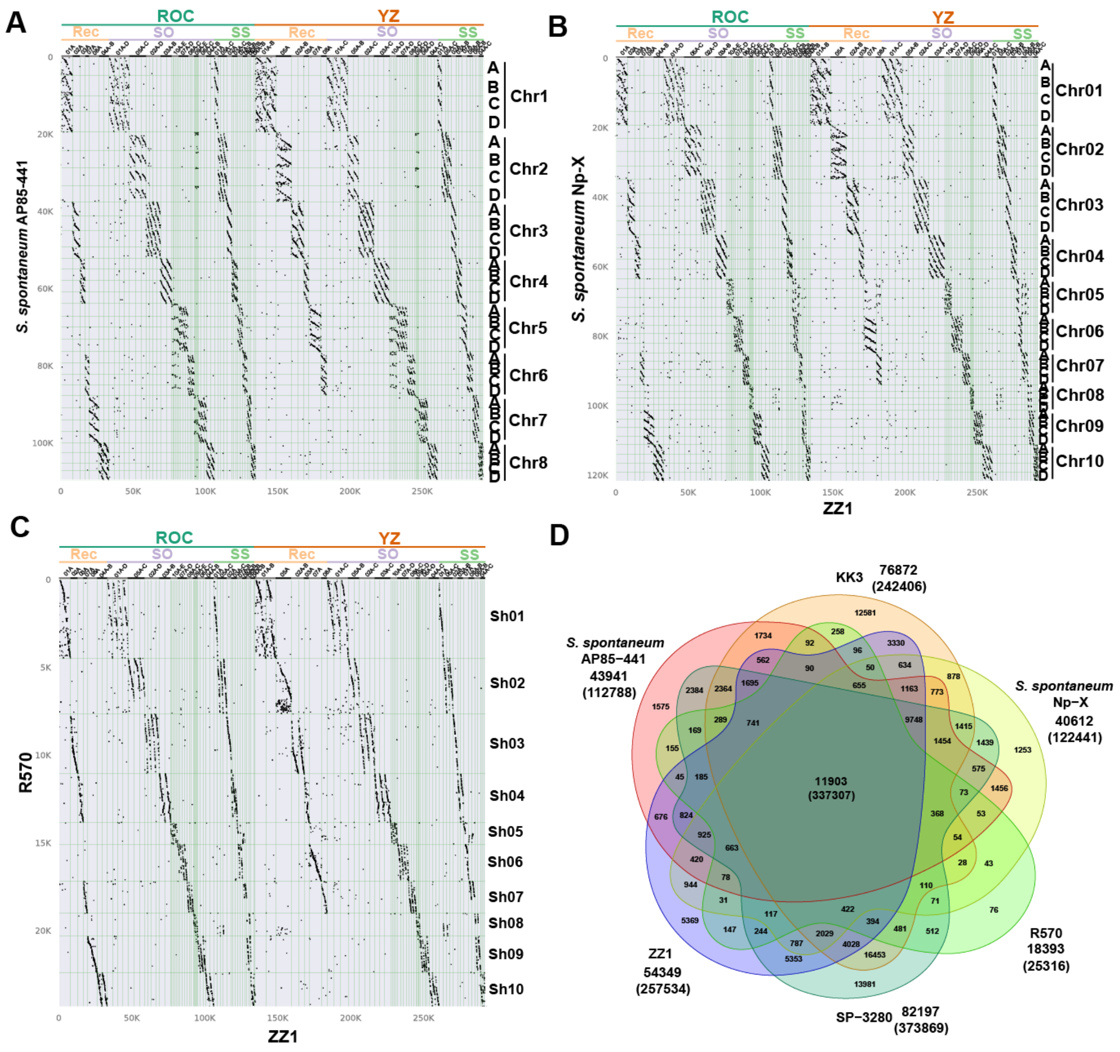

Supplementary Figure 3. The comparison of genome synteny and ortholog gene cluster between ZZ1 and published sugarcane genomes. The genome synteny between ZZ1 and *S. spontaneum* AP85-441(A), *S. spontaneum* Np-X(B), and R570(C). (D) The venn diagram illustrated the ortholog gene clusters among ZZ1 and published sugarcane genomes.

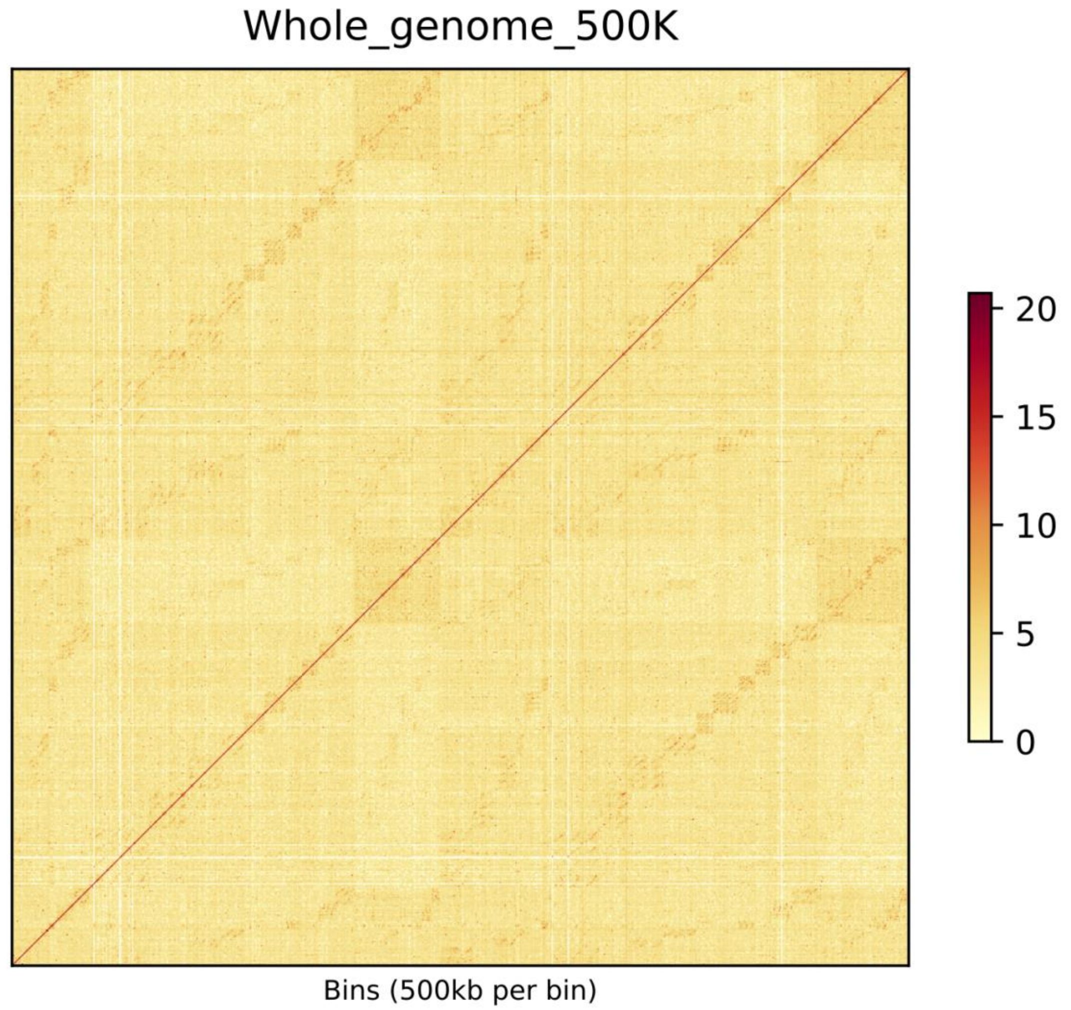

Supplementary Figure 4. Genome-wide analysis of chromatin interactions at 500-kb resolution in ZZ1 genome.

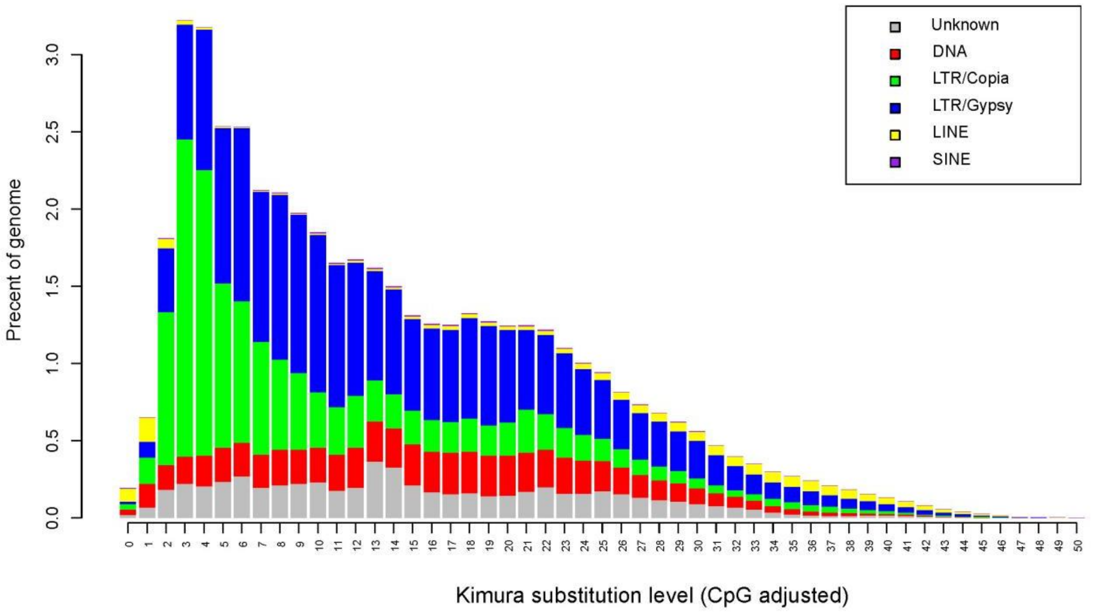

Supplementary Figure 5. Composition of TEs and Kimura distance in ZZ1 genome.

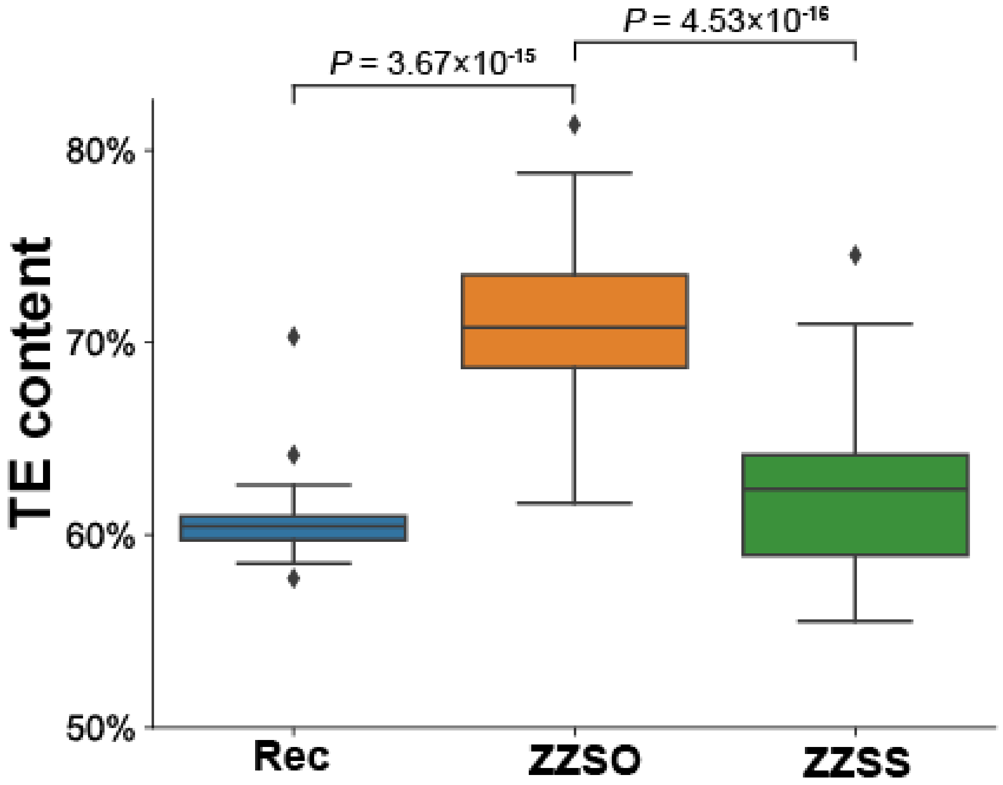

Supplementary Figure 6. The comparison of TE content between the Rec, ZZSO, ZZSS region of ZZ1 genome. Significance was tested with t-test. In the box plots, central line: median values; bounds of the box: 25th and 75th percentile.

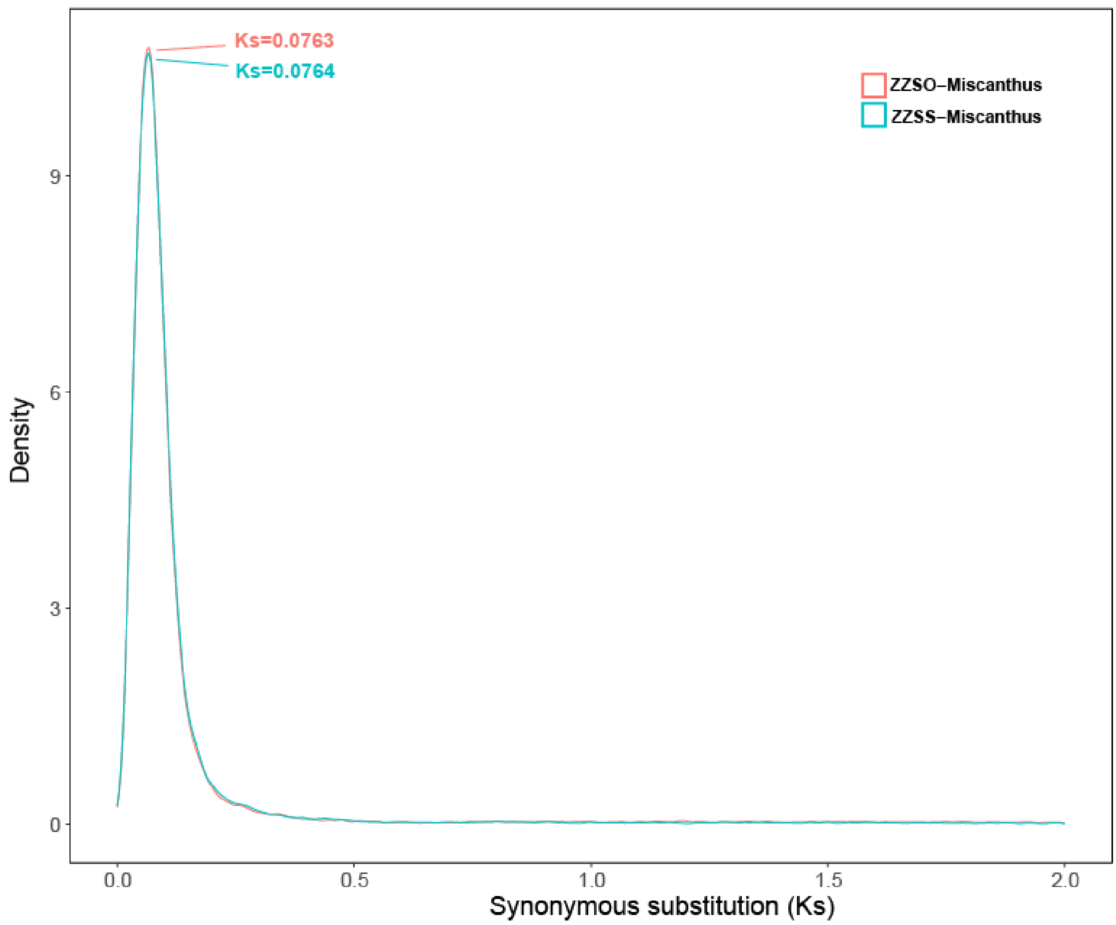

Supplementary Figure 7. The comparison of synonymous substitution (Ks) between subgenomes of ZZ1 and Miscanthus.

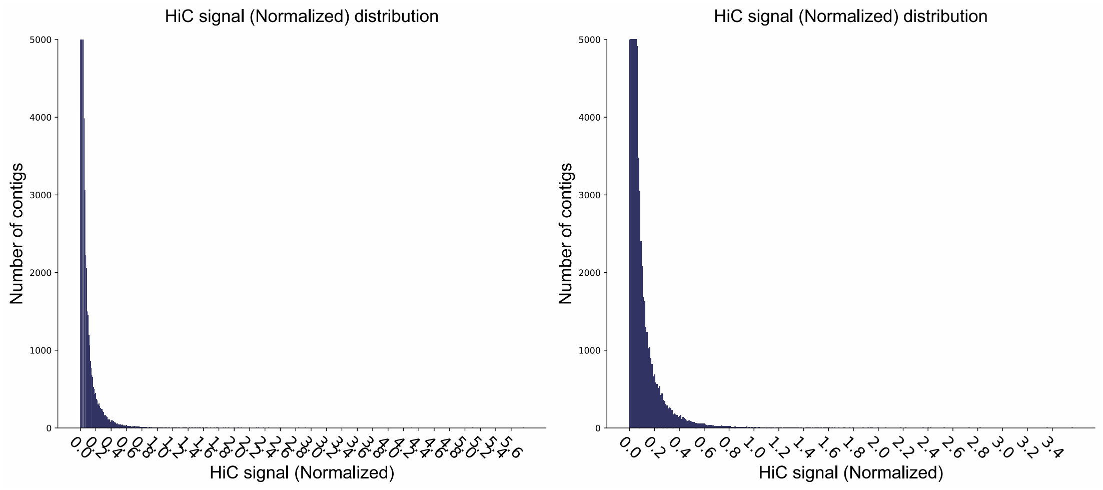

Supplementary Figure 8. Normalized HiC signal distribution in ROC (left) and YZ (right).

Supplementary references

1. Garsmeur, O. *et al.* A mosaic monoploid reference sequence for the highly complex genome of sugarcane. *Nature Communications* 9, 2638 (2018).
2. Shearman, J.R. *et al*. A draft chromosome-scale genome assembly of a commercial sugarcane. *Scientific Reports* 12, 20474 (2022).
3. Souza, G.M. *et al*. Assembly of the 373k gene space of the polyploid sugarcane genome reveals reservoirs of functional diversity in the world's leading biomass crop. *GigaScience* 8, giz129 (2019).
4. Zhang, Q. *et al*. Genomic insights into the recent chromosome reduction of autopolyploid sugarcane *Saccharum spontaneum*. *Nature Genetics* 54, 885-896 (2022).
5. Zhang, J. *et al*. Allele-defined genome of the autopolyploid sugarcane *Saccharum spontaneum* L. *Nature Genetics* 50, 1565-1573 (2018).
