# Supplementary material for: A chromosomal-scale genome assembly of modern cultivated hybrid sugarcane provides insights into origination and evolution
Source: Nat Commun. 2024 Apr 8;15:3041. doi: 10.1038/s41467-024-47390-6 (PMC11001919; doi:10.1038/s41467-024-47390-6)
Supplement: Supplementary file 3 — Description of Additional Supplementary Files [file 41467_2024_47390_MOESM3_ESM.pdf]

Description of Additional Supplementary Files

File Name: Supplementary Data 1
Description: Allele-defined annotation of ZZ1 genome
