# Supplementary material for: A chromosomal-scale genome assembly of modern cultivated hybrid sugarcane provides insights into origination and evolution
Source: Nat Commun. 2024 Apr 8;15:3041. doi: 10.1038/s41467-024-47390-6 (PMC11001919; doi:10.1038/s41467-024-47390-6)
Supplement: Supplementary file 5 — Reporting Summary [file 41467_2024_47390_MOESM5_ESM.pdf]

nature portfolio

Corresponding author(s): Muqing Zhang, Xingtan Zhang, Baoshan Chen

Last updated by author(s): Mar 10, 2024

Reporting Summary

Nature Portfolio wishes to improve the reproducibility of the work that we publish. This form provides structure for consistency and transparency in reporting. For further information on Nature Portfolio policies, see our Editorial Policies and the Editorial Policy Checklist.

Statistics

For all statistical analyses, confirm that the following items are present in the figure legend, table legend, main text, or Methods section.

n/a	Confirmed	
☐	☒	The exact sample size (*n*) for each experimental group/condition, given as a discrete number and unit of measurement
☐	☒	A statement on whether measurements were taken from distinct samples or whether the same sample was measured repeatedly
☐	☒	The statistical test(s) used AND whether they are one- or two-sided<br>*Only common tests should be described solely by name; describe more complex techniques in the Methods section.*
☒	☐	A description of all covariates tested
☒	☐	A description of any assumptions or corrections, such as tests of normality and adjustment for multiple comparisons
☒	☐	A full description of the statistical parameters including central tendency (e.g. means) or other basic estimates (e.g. regression coefficient) AND variation (e.g. standard deviation) or associated estimates of uncertainty (e.g. confidence intervals)
☐	☒	For null hypothesis testing, the test statistic (e.g. *F*, *t*, *r*) with confidence intervals, effect sizes, degrees of freedom and *P* value noted<br>*Give P values as exact values whenever suitable.*
☒	☐	For Bayesian analysis, information on the choice of priors and Markov chain Monte Carlo settings
☒	☐	For hierarchical and complex designs, identification of the appropriate level for tests and full reporting of outcomes
☒	☐	Estimates of effect sizes (e.g. Cohen's *d*, Pearson's *r*), indicating how they were calculated

Our web collection on statistics for biologists contains articles on many of the points above.

Software and code

Policy information about availability of computer code

Data collection	PacBio CCS (Circular Consensus Sequencing) reads were collected from single-molecule real-time (SMRT) cells on PacBio Sequel II platform; Pair-end sequencing reads, Hi-C reads and transcriptome sequencing reads were collected from the Illumina NoveSeq platform.
Data analysis	All softwares and corresponding versions have been well described in the method. The software used in this study: Cutadapt v2.0, HISAT2 v2.1.0, Cufflinks v2.2.0, Hifiasm v0.11-r302; BWA v0.7.15; ALLHiC v0.1; BLASTN v2.7.1; BUSCO V3.0.2; popCNV (https://github.com/sc-zhang/popCNV); minimap2; CATG v0.1(https://gitee.com/tanger-lab_enterprise/CATG); Merqury v0.1; RepeatModeler v2.0.1; RepeatMasker v4.0; RECON v1.08; RepeatScout v1.0.5; GETA v0.1; augustus v3.3.2; trimmomatic v0.38; HiSAT2 v2.1.0; genewise v2.4.1; TransDecoder v5.7.1; GetaFilter (A self-written perl pipeline); Khaper v0.1; MCScanX (http://chibba.pgml.uga.edu/mcscan2/); bowtie2 v2.3.4.3; Trinity v2.3.2; R/QTL v1.39; MCscan (http://github.com/tanghaibao/jcvi/wiki/MCsacn-(Python-version)); HMMER v3.0.

For manuscripts utilizing custom algorithms or software that are central to the research but not yet described in published literature, software must be made available to editors and reviewers. We strongly encourage code deposition in a community repository (e.g. GitHub). See the Nature Portfolio guidelines for submitting code & software for further information.

Data

Policy information about availability of data

All manuscripts must include a data availability statement. This statement should provide the following information, where applicable:

- Accession codes, unique identifiers, or web links for publicly available datasets
- A description of any restrictions on data availability
- For clinical datasets or third party data, please ensure that the statement adheres to our policy

Relevant data supporting the findings of this study are available in this article and its Supplementary Information files. The raw data, genome assemblies, and annotation data generated in this study have been deposited in the China National Center for Bioinformation Genome Warehouse under accession code GWHEQVP00000000 (https://ngdc.cncb.ac.cn/gwh/Assembly/83532/show). The SP80-3280 genome data were downloaded from NCBI under accession number GCA_009173535.1 (https://www.ncbi.nlm.nih.gov/search/all/?term=GCA_009173535.1); KK3 were downloaded from NCBI under accession number JALQSO000000000 (https://www.ncbi.nlm.nih.gov/nuccore/JALQSO000000000.1/); R570 were downloaded from sugarcane genome hub (http://sugarcane-genome.cirad.fr); AP85-441 were downloaded from NCBI under accession number QVOL00000000 (https://www.ncbi.nlm.nih.gov/datasets/genome/GCA_003544955.1/); Np-X were deposited into the Sequence Read Archive (under BioProject accession PRJNA721787) (https://www.ncbi.nlm.nih.gov/bioproject/PRJNA721787/); LA-Purple were available from NCBI with the same accession number and under Bioproject accession PRJNA744175 (https://www.ncbi.nlm.nih.gov/bioproject/PRJNA744175/); The RNA-Seq raw data used in this study have been deposited in NCBI under accession number SRR28212565 to SRR28212604 (https://submit.ncbi.nlm.nih.gov/subs/sra/SUB14287022/overview); Rice, Sorghum and maize information was collected from the article49-51. Source data are provided in this paper. The public databases used in this study: Pfam database (http://ftp.ebi.ac.uk/pub/databases/Pfam/current_release); UniProt database (http://www.uniprot.org/); GO database (http://geneontology.org/); KEGG database (https://www.kegg.jp); TrEMBL (http://www.expasy.org/sprot); Swiss-Prot database (http://www.uniprot.org/downloads); and KOG database (ftp://ftp.ncbi.nih.gov/pub/COG/KOG/kyva).

Research involving human participants, their data, or biological material

Policy information about studies with human participants or human data. See also policy information about sex, gender (identity/presentation), and sexual orientation and race, ethnicity and racism.

Reporting on sex and gender	not applicable
Reporting on race, ethnicity, or other socially relevant groupings	not applicable
Population characteristics	not applicable
Recruitment	not applicable
Ethics oversight	not applicable

Note that full information on the approval of the study protocol must also be provided in the manuscript.

Field-specific reporting

Please select the one below that is the best fit for your research. If you are not sure, read the appropriate sections before making your selection.

☒ Life sciences ☐ Behavioural & social sciences ☐ Ecological, evolutionary & environmental sciences

For a reference copy of the document with all sections, see nature.com/documents/nr-reporting-summary-flat.pdf

Life sciences study design

All studies must disclose on these points even when the disclosure is negative.

Sample size	Samples were selected to have enough representation from cultivated and wild accessions to query meaningful and decisive conclusion.
Data exclusions	No data were excluded.
Replication	For RNA-seq of leaf, stem, root and bud, three replicates for each samples were performed. The FISH experiments were repeated independently at least three times with similar results.
Randomization	This is not relevant to our study as all of the analyses followed published and well-eatablished methods.
Blinding	The investigators were blinded to group allocation during data collection.

Reporting for specific materials, systems and methods

We require information from authors about some types of materials, experimental systems and methods used in many studies. Here, indicate whether each material, system or method listed is relevant to your study. If you are not sure if a list item applies to your research, read the appropriate section before selecting a response.

Materials & experimental systems

n/a	Involved in the study
☒	☐ Antibodies
☒	☐ Eukaryotic cell lines
☒	☐ Palaeontology and archaeology
☒	☐ Animals and other organisms
☒	☐ Clinical data
☒	☐ Dual use research of concern
☐	☒ Plants

Methods

n/a	Involved in the study
☒	☐ ChIP-seq
☐	☒ Flow cytometry
☒	☐ MRI-based neuroimaging

Plants

Seed stocks	The plant Zhong Zhe No. 1 (ZZ1) was cultivated in the greenhouse at Guangxi University, Nanning City, Guangxi Zhuang autonomous region, China (108.33 °E, 22.84 °N). The young leaves from the same individual were flash-frozen in liquid nitrogen and stored at -80 ºC until DNA extraction and genome sequencing.
Novel plant genotypes	not applicable
Authentication	The Guangxi university is providing plant materials. The variety was registered and approved by the Ministry of Agriculture and Rural Affairs of China, in 2014.

Flow Cytometry

Plots

Confirm that:

☐ The axis labels state the marker and fluorochrome used (e.g. CD4-FITC).

☐ The axis scales are clearly visible. Include numbers along axes only for bottom left plot of group (a 'group' is an analysis of identical markers).

☒ All plots are contour plots with outliers or pseudocolor plots.

☐ A numerical value for number of cells or percentage (with statistics) is provided.

Methodology

Sample preparation	Reference (the seeds of maize B73 and tomato) were obtained from Kunming Institute of Botany, Chinese Academy of Sciences, and the test materials were the young leaves (seedling stage, 1 month) 'ZZ1'. (i) Place a small amount of plant seed (typically 20 mg) in the center of a plastic Petri dish. (ii) Add 1 ml ice-cold nuclei isolation buffer ((mGb buffer: 45 mM MgCl2·6H2O, 20 mM MOPS, 30 mM sodium citrate, 1% (W/V) PVP 40, 0.2% (v/v) Tritonx-100, 10 mM Na2EDTA, pH 7.0) to the Petri dish. (iii) Chop the tissue immediately in the buffer with a new razor blade or a sharp (disposable) scalpel. (iv) Mix the homogenate by pipetting up and down for several times (avoid air bubbles). (v) Filter the homogenate through a 42-µm nylon mesh into a labeled sample tube. (vi) Add stock solution of a DNA fluorochrome and shake gently. PI is typically used at 50 mg/ ml simultaneously with RNase at 50 mg/ ml. (vii) Incubate the sample on ice before analysis (a few minutes to 1 h), with occasional shaking. (viii) FCM measures the relative fluorescence of the stained nuclei.
Instrument	BD FACS calibur
Software	The emission fluorescence intensity of propidium iodide was detected by 488 nm blue light excitation, and use Modifit3.0 analysis software for graph analysis.
Cell population abundance	The coefficient of variation (CV%) was controlled within 5% and 5000-10000 particles were collected for each detection.
Gating strategy	Density maps are used to distinguish between target cell clusters and fragments, with higher density areas having higher cell concentrations, and these areas are generally delineated as target cell clusters. The horizontal and vertical coordinates are FSC-H, SSC-H, respectively. By adjusting the photomultiplier voltage of FSC and SSC, the target cells can be selected from the FSC-A/SSC-A scatter plot. After logarithmic amplification, the nuclei are clustered together and in the center of the diagram, and the target nuclei are circled by setting a gating strategy.

☒ Tick this box to confirm that a figure exemplifying the gating strategy is provided in the Supplementary Information.
