# Supplementary material for: A chromosomal-scale genome assembly of modern cultivated hybrid sugarcane provides insights into origination and evolution
Source: Nat Commun. 2024 Apr 8;15:3041. doi: 10.1038/s41467-024-47390-6 (PMC11001919; doi:10.1038/s41467-024-47390-6)
Supplement: Supplementary file 2 — Peer Review File [file 41467_2024_47390_MOESM2_ESM.pdf]

Peer Review File

A chromosomal-scale genome assembly of modern cultivated hybrid sugarcane provides insights into origination and evolution

Open Access This file is licensed under a Creative Commons Attribution 4.0 International License, which permits use, sharing, adaptation, distribution and reproduction in any medium or format, as long as you give appropriate credit to the original author(s) and the source, provide a link to the Creative Commons license, and indicate if changes were made. In the cases where the authors are anonymous, such as is the case for the reports of anonymous peer reviewers, author attribution should be to 'Anonymous Referee' followed by a clear attribution to the source work. The images or other third party material in this file are included in the article's Creative Commons license, unless indicated otherwise in a credit line to the material. If material is not included in the article's Creative Commons license and your intended use is not permitted by statutory regulation or exceeds the permitted use, you will need to obtain permission directly from the copyright holder. To view a copy of this license, visit http://creativecommons.org/licenses/by/4.0/.

Reviewers' Comments:

Reviewer #1:
Remarks to the Author:

See attached .doc

As the first report of a phased chromosome level assembly of a complex hybrid sugarcane genome, this is an important contribution to the sugarcane community. The authors are to be congratulated for overcoming the technical challenges in assembling this complex polyploid genome.

Two specific questions and comments:

Lines 96/97: The texts mentions 'artificial sequences' and 'artificial k-mers'. Does this refer to kimeric sequences generated during sequencing – i.e. artifacts? Is 'artificial' the best word to use here? Or artifactual sequences?

Lines 222/223: Its seems that the inference here is that because the variety ZZ1 has more sugar transport gene members than the reference genomes of S.spontaneum (Clone AP85-441) and S.officinarum (LA Purple), that gene expansion occurred during breeding. It is known, however, that at least 19 different S.officinarum clones and a range of S.spontaneum, S,barberi and S.sinense clones contribute to the genealogy of modern hybrids. It is likely that the additional gene members found in ZZ1 are derived from other ancestral clones of SO and SS, and not due to gene expansion during the several generations of breeding that led to variety ZZ1.

One general comment relating to genome nomenclature and its implications for the sugarcane genomics community.

It has been recognized from some time that the origin of modern sugarcane hybrids are S.spontaneum with x=8 and S.officinarum with x=10, and that structural rearrangements between the genomes of the two species will lead to issues in chromosome nomenclature. Without standardization, this will lead to confusion in the literature. For example, in this submission, the nomenclature of LA Purple was used (based on sorghum chrm nomenclature), with the results that Chr5 and Chr 8 are 'missing' for SS, while Chr 9 and Chr10 are 'present' for SS – whereas the published genome for SS (Zhang et al, 2018), names SS chromosomes for 1 to 8 – i.e. there is a lack of correspondence between the chromosome nomenclature of Zhang 2018, with this submission.

The proposed solution to this problem was published by Garsmeur et al 2018, by de-linking Saccharum chromosome nomenclature from Sorghum, and creating a specific Saccharum nomenclature. The Garsmeur et al 2018 proposal is summarized below.

S.off	1	2	3	4	5	6	7	8	9	10
S.spont	1	2	3	4	5 (5/9)	6 (6/9)	7 (7/10)	8 (8/10)		
Sorghum	1	3	4	10	2	9	6	7	8	5

By adopting this nomenclature, future SS genomes can be named 1 to 8 and future hybrid genomes named 1 to 10, without confusion. It is strong suggested to the authors to consider adopting the nomenclature proposed by Garsmeur 2018 in order to facilitate and standardize sugarcane chromosome nomenclature.

d

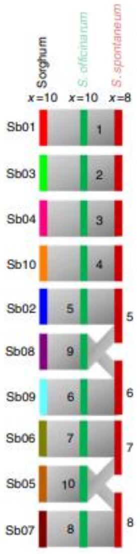
Sorghum
S. officinarum
S. spontaneum
x=10
x=10
x=8
Sb01
Sb03
Sb04
Sb10
Sb02
Sb08
Sb09
Sb06
Sb05
Sb07
1
2
3
4
5
9
6
7
10
8
5
6
7
8

Reviewer #2:
Remarks to the Author:
Overall evaluation
==================
This manuscript presents a new exciting genome assembly and analysis of the species Saccharum officinarum. This species has a quite complex genome and previous assemblies are incomplete. The latest advances in the sequencing technologies have allowed to the authors to produce an excellent genome assembly. Nevertheless, there are several points that the authors should resolve: 1- The lack of important details in the material and methods, which makes difficult an full assessment; 2- A deeper discussion on the results with a better contextualization and the inclusion of many articles about the complex sugarcane genome; 3- An extended analysis of the genome structure and evolution of this species; 4- Revision of the format of the manuscript including the coherence between text and tables. So overall, it is a worthy to read manuscript. For sure, something publishable from which the community will benefit once the major concerns have been resolved.

Point-By-Point Manuscript Evaluation:
==
A. Summary of the key results

The manuscript titled "A chromosomal-scale genome assembly of modern cultivated hybrid sugarcane provides insights into origination and evolution" describes a new genome assembly and analysis of the complex polyploid genome of sugarcane (Saccharum officinarum) including the transcriptomic analysis of several developmental stages. The key results can be summarized as:
• Sequencing, assembly, and annotation of the sugarcane accession ZZ1. The assembly produced has a size of 10.4 Gb. 87% of the assembly was anchored into 114 chromosomes. 66.5% of the genome was annotated as repetitive elements. The genome annotation delivered 257,534 protein-coding gene models. The origin of 68,509 gene models were identified according the three different sub-genomes.
• The cytogenetic characterization of the sugarcane accession ZZ1 described a genome of approx. 9 Gb and 114 chromosomes of which 68 chromosomes derived from S. officinarum, 31 from S. spontaneum, and 15 representing recombination between sub-genomes.
• The authors did not find sub-genome dominance for gene expression, although they found that 7.1% of the gene expression was biased to S. officinarum, and 3.0% to S. spontaneum.
• The pathogen response to Pokkah boeng disease is driven mainly by the S. officinarum, sub-genome rather than the S. spontaneum according to a transcriptomic analysis. Nevertheless, the non-overlapping function of the genes of each sub-genome indicates a possible cooperative response between them.

B. Originality and significance: if not novel, please include reference
----
This manuscript presents a new exciting assembly of the complex sugarcane genome. There have been several genome assemblies of Saccharum spontaneum (Zhang et al., 2018, Zhang et al., 2021) where the origin of this species is discussed. There also some fragmented genome assemblies for S. officinarum (Garsmeur et al., 2018, Souza et al., 2019), but the incompleteness of the assemblies limited the analysis.

C. Data & methodology: validity of approach, quality of data, quality of presentation.

The methodology used looks adequate although there are many missing details in the Material & Methods section that makes difficult a full assessment. For example, the HMW DNA extraction method has not been described in detail (there is only a link to 10X genomics), so it is a gap in the assessment of how the high-quality SMRTbell libraries (30-50 kb) were produced. RNA extraction method has not been specified either, or any of the versions of the program used. The Hi-C map is not very helpful to identify the possible pseudo-chromosomes. I recommend having in the supplementary a figure with the chromosomes with assigned bins and because there are many of them, split the

figure in 6-7 so the readers could inspect the results in more details. In this sense, the authors should also report the number of contacts produced by the Hi-C map. The assessment of the quality of the assembly is partial. The authors have used Merqury but not reported the results about consensus quality and completeness (at least not in the result section). No assessment of the completeness of the LTR elements (e.g., using LTR_retriever). The contamination screening was performed only by Blast against bacterial genomes, but not, for example, fungi. The use of Blobtools is recommended in this case. There are some parameters missing in the gene model annotation that could be useful to assess the quality such as the number of single exon gene, length of the gene models, numbers of UTR annotated, number of genes supported by RNA-Seq data… No description of the RNA-Seq analysis, including the number of replicates per sample. The quality of the genome assembly should be described using the standards of the Earth Biogenome Project (https://www.earthbiogenome.org/assembly-standards).

D. Appropriate use of statistics and treatment of uncertainties
----
Yes, in some extension (see previous section).

E. Conclusions: robustness, validity, reliability

The conclusions are valid according to the results presented, but the lack of details makes difficult a full assessment (for example, for the expression analysis, it needs to be assumed that the authors used three independent replicates of plants challenged by the pathogen and with clear phenotypes of the infection… none of these details are in the material and methods).

F. Suggested improvements: experiments, data for possible revision.

The manuscript lacks important details in the material and method section.

The discussion also should be improved. There is not an extensive discussion of the polyploid nature of the genome in the context of origin (e.g., no dating, or comparison with the results of the Saccharum spontaneum articles (Zhang et al., 2018, Zhang et al., 2021)). The discussion is more a summary rather than a contextualization in which different results are compared with previously published results driving to new hypothesis and insights.

The authors did an excellent characterization of the origin of the different sub-genomes. Nevertheless, I think that there are several pieces that it could be interesting to explore about the history of this complex genome:

- Tracing of the maternal inherence of the organelle genomes through the different rounds of hybridization and polyploidization. The authors did not mention anything about the chloroplast and mitochondria origin of the ZZ1 accession. The suppl. figure 1 states ROC1 as the maternal donor but it will be interesting to know if they derived from S. robustum or S. officinarum.
- Dating of speciation and hybridization events. The authors did estimate the divergency between the different sub-genomes using the synonymous substitution (Ks) of the ortholog pairs of SO-ZZSO, SS-ZZSS, ZZSS-ZZSO, and SO-SS. It will add value to the results and the discussion to see some age estimation associated to this Ks ratio values. The authors could even use the Miscanthus genomes (Zhang et al. 2021) to date the possible divergency dates between the different sub-genomes (Miscanthus- Saccharum split dated as 7.9 MYA according https://timetree.org/).
- Homologous exchange transposition (HET) characterization. There is not an extensive analysis of the regions of the genome that present HET. It could be interesting to have a deeper look of this part with some questions (for example, are the HET regions rich in TEs favoring the non-reciprocal exchanges?).

The manuscript needs a format revision. For example, the first time that you introduce a species name, it needs to be used as complete name (e.g., Saccharum officinarum). Then, it can be abbreviated using only the first letter of the genus (as S. officinarum). In the line 182, the species S.

officinarum is mistyped as S. officianrum and in the line 210 as Sacchuarm. In some paragraphs, the authors use the species names (S. spontaneum and S. officinarum) while in other ones they use just an abbreviation (SS or SO). The same criteria should be used for the whole text. It also needs a consistency revision between the numbers presented in the text and the tables. For example, in the line 141 is described that 257,534 protein-coding gene models were identified. Nevertheless, in the table 1, this number goes to 370,103.

G. References: appropriate credit to previous work?

Yes, in some extend, but I found some references missing like the one for the reference genome S. officinarum LA-purple.

H. Clarity and context: lucidity of abstract/summary, appropriateness of abstract, introduction and conclusions.

The abstract is adequate, and clear.

Revised by Aureliano Bombarely on Oct. 31st, 2023.

Reviewer #3:
Remarks to the Author:
In the manuscript entitled "A chromosomal-scale genome assembly of modern cultivated hybrid sugarcane provides insights into origination and evolution," the authors describe a haplotype resolved chromosome-scale assembly of the ZZ1 cultivar of sugarcane. The authors describe how they assembled the highly complex ZZ1 genomes using long and short reads couple to HiC. In the abstract the authors claimed they developed new methods ("…series of bioinformatics approaches we developed…"), but it was unclear from the description that any new/novel assembly techniques were used in the process. The authors then go on to look at homoeolog expression dominance and conclude there is none in ZZ1. In addition, the authors look at expression after treatment with the fusarium pest Pokkah boeng, and report that it may be a combination of the subgenomes that provides resistance (based on expression). The manuscript reads much like a genome announcement and only addresses the origin and evolution of the sugarcane genome through the comparison and expression of alleles. Since this is primarily a manuscript about an improved sugarcane genome assembly, the genome needs to be available to the reviewers to assess; impossible to review the content otherwise. Also, the authors do very little to no comparison with the sugarcane genome assemblies that are currently available.

Below are small comments concerning grammar content and writing.

If you are going to use "Rec" to represent the recombined chromosomes, then use it consistently throughout.

Please use ZZ1 or ZZ consistently throughout as the name of the cultivar.

Would be great to add a bit in the introduction about Pokkah boeng (and disease resistance in sugarcane) in the introduction.

Line 45 "….leading to a colossal genome size of ~10 Gb."
It is large but not "colossal;" gymnosoperms and above 40 Gb is colossal-this is just a large genome for a plant.

Lines 133-135: "The comprehensive approach was used to detect allele genes within the ultra-

complex genome based on the monoploid genomes and annotated genes of two ancestors (see Method)."
Sentence does not make sense.

In the section from lines 156-182, the authors use the word "decents;" do the authors mean descendants? It could also be used to mean descent (trajectory) but that does not fit well in the context of what is being said.

Lines 211-213: "Sugar transporter family: A total of 130 genes, including 1,166 alleles, likely belong to the members of the sugar transporter superfamily consisting of PLT, VGT, SFP, TMT, STP, pGlcT, INT, SUT, and SWEET subfamily."
Spell out

Line 219: "…and their reconstruction in the leaf of the different development stage…"
Not sure what this means; please clarify.

Lines 221-223: "In addition, the PLT, TMT, and SWEET families had more gene members than S. spontaneum, S. officinarum, and their relative species, suggesting that gene expansion occurred in these three families during the breeding of ZZ."

Lines 225-231: The section on NBS has very little content.

Line 240: "lineages showed dominant expression (LDE)"
Does LDE stand for Lineage Dominant Expression? If so they this sentence needs to be reconstructed to make that clear.

Line 255: "…genes that showed up-regulated in SO…"
Maybe should read, "…genes that were up-regulated in SO…"

REVIEWER COMMENTS

Reviewer #1 (Remarks to the Author):

As the first report of a phased chromosome level assembly of a complex hybrid sugarcane genome, this is an important contribution to the sugarcane community. The authors are to be congratulated for overcoming the technical challenges in assembling this complex polyploid genome.

Response: Thanks for your kind words. We appreciate your recognition of our work as the first report of a phased chromosome-level assembly of a complex hybrid sugarcane genome. We are grateful for the opportunity to contribute to the sugarcane community and overcome the technical challenges.

Two specific questions and comments:

Lines 96/97: The texts mentions 'artificial sequences' and 'artificial k-mers'. Does this refer to kimeric sequences generated during sequencing – i.e. artifacts? Is 'artificial' the best word to use here? Or artifactual sequences?

Response: The 'artificial sequences' was one type of error assembly derived from the primarily assembled contigs, and it could be identified by performing K-mers analysis on the Mercury program. In our study, these sequences contained a large proportion (> 40%) of K-mers present only in assembly but absent in the sequencing reads were detected as 'artificial sequences', and then removed.

Yes, the word "artifactual" seems more suitable. We have revised the resubmitted version. Thank you!

Lines 222/223: Its seems that the inference here is that because the variety ZZ1 has more sugar transport gene members than the reference genomes of S.spontaneum (Clone AP85-441) and S.officinarum (LA Purple), that gene expansion occurred during breeding. It is known, however, that at least 19 different S.officinarum clones and a range of S.spontaneum, S,barberi and S.sinense clones contribute to the genealogy of modern hybrids. It is likely that the additional gene members found in ZZ1 are derived from other ancestral clones of SO and SS, and not due to gene expansion during the several generations of breeding that led to variety ZZ1.

Response: Thank you for your perspective, which is indeed insightful. We fully agree with your viewpoint. The breeding process of modern cultivated sugarcane is complex, involving the incorporation of lineages from multiple clones. The genes associated with important agronomic traits are likely derived from the intermingling of various lineages. However, gene family expansion may contribute to increased gene family members. This is because the formation of hybrid species can trigger genetic evolutionary mechanisms, including transposon jumping, leading to genome expansion (Ungerer et al., 2006; Ishikawa et al., 2009). Of course, this requires more data to explore next. Therefore, in light of these two viewpoints, we have incorporated them into the revised manuscript.

Ungerer MC, Strakosh SC, Zhen Y. Genome expansion in three hybrid sunflower species is associated with retrotransposon proliferation. Curr Biol. 2006 Oct 24;16(20):R872-R873.

Ishikawa R, Kinoshita T. Epigenetic programming: the challenge to species hybridization. Mol Plant. 2009 Jul;2(4):589-599.

One general comment relating to genome nomenclature and its implications for the sugarcane genomics community.

It has been recognized from some time that the origin of modern sugarcane hybrids are *S.spontaneum* with x=8 and *S.officinarum* with x=10, and that structural rearrangements between the genomes of the two species will lead to issues in chromosome nomenclature. Without standardization, this will lead to confusion in the literature. For example, in this submission, the nomenclature of LA Purple was used (based on sorghum chrm nomenclature), with the results that Chr5 and Chr 8 are 'missing' for SS, while Chr 9 and Chr10 are 'present' for SS – whereas the published genome for SS (Zhang et al, 2018), names SS chromosomes for 1 to 8 – i.e. there is a lack of correspondence between the chromosome nomenclature of Zhang 2018, with this submission.

The proposed solution to this problem was published by Garsmeur et al 2018, by de-linking Saccharum chromosome nomenclature from Sorghum, and creating a specific Saccharum nomenclature. The Garsmeur et al 2018 proposal is summarized below.

S.off 1 2 3 4 5 6 7 8 9 10

S.spont 1 2 3 4 5 (5/9) 6 (6/9) 7 (7/10) 8 (8/10)

Sorghum 1 3 4 10 2 9 6 7 8 5

By adopting this nomenclature, future SS genomes can be named 1 to 8 and future hybrid genomes named 1 to 10, without confusion. It is strong suggested to the authors to consider adopting the nomenclature proposed by Garsmeur 2018 in order to facilitate and standardize sugarcane chromosome nomenclature.

See attached .doc

Response: Thank you for your highly professional suggestions. Your advice holds great significance in advancing the field of sugarcane research. We are pleased to adopt the proposed nomenclature and have corrected the text and figures throughout the manuscript accordingly. Additionally, to facilitate a faster and more accurate understanding of the chromosomal correspondence between hybrid sugarcane and previously published works, including Garsmeur et al. 2018, Zhang et al. 2018, Zhang et al. 2022, and sorghum, we have included a chromosome numbering table as an attachment to the revised manuscript. The table is as following:

Supplementary Table 1. Correspondence table of sugarcane chromosome nomenclature

**Sorghum**	***S. spontaneum* Np-X**	***S. spontaneum* AP85-441**	**R570**			**ZZ1**		
			**STP**	**SS**	**SO**	**Chr. groups**	**SS**	**SO**
**Sb01**	Chr01	Chr1	Sh01	1	1	Chr01	Ss-Chr01	So-Chr01
**Sb02**	Chr02		Sh02		5	Chr02		So-Chr05
		Chr2		5			Ss-Chr05	
**Sb08**	Chr08		Sh08		9	Chr08		So-Chr09
		Chr7		6			Ss-Chr06	
**Sb09**	Chr09		Sh09		6	Chr09		So-Chr06
**Sb06**	Chr06		Sh06		8	Chr06		So-Chr07
		Chr5		7			Ss-Chr07	
**Sb05**	Chr05		Sh05		10	Chr05		So-Chr10
		Chr6		8			Ss-Chr08	
**Sb07**	Chr07		Sh07		8	Chr07		So-Chr08
**Sb03**	Chr03	Chr3	Sh03	2	2	Chr03	Ss-Chr02	So-Chr02
**Sb04**	Chr04	Chr4	Sh04	3	3	Chr04	Ss-Chr03	So-Chr03
**Sb10**	Chr10	Chr10	Sh10	4	4	Chr10	Ss-Chr04	So-Chr04

Reviewer #2 (Remarks to the Author):

Overall evaluation

==================

This manuscript presents a new exciting genome assembly and analysis of the species Saccharum officinarum. This species has a quite complex genome and previous assemblies are incomplete. The latest advances in the sequencing technologies have allowed to the authors to produce an excellent genome assembly. Nevertheless, there are several points that the authors should resolve: 1- The lack of important details in the material and methods, which makes difficult an full assessment; 2- A deeper discussion on the results with a better contextualization and the inclusion of many articles about the complex sugarcane genome; 3- An extended analysis of the genome structure and evolution of this species; 4- Revision of the format of the manuscript including the coherence between text and tables. So overall, it is a worthy to read manuscript. For sure, something publishable from which the community will benefit once the major concerns have been resolved.

Response: Thank you for your valuable feedback on our manuscript. We truly appreciate your kind words, as they are encouraging, especially considering the exhaustive process of assembling such a complex genome. Rest assured, we are fully dedicated to addressing the concerns you raised and making the necessary revisions to enhance the quality of the manuscript further. Your thoughtful evaluation is greatly appreciated, and we are grateful for your support throughout this process.

1- The lack of important details in the material and methods, which makes difficult a full assessment;

Response: Thanks for your comments. We added more details in the material and methods section. The supplemented content is as following:

Line 461-479:

Sample preparation and genome sequencing

RNA extraction, sequencing, and profiling. Spore suspension of *Ssc* (the causative agent of smut) (1×10^6 spores/mL) was inoculated at the growth point of ZZ1, at the young roots and buds, respectively. Inoculated plants were placed in a constant temperature incubator at 28° C in a medium moisturizing culture. Smut group samples were collected at 5d and 20d after inoculation, with water as a control treatment. In addition, the different tissues and different developmental stages were collected from ZZ1, including 'leaf in pre-mature stage (ZBL), stem in pre-mature stage (ZBS), leaf in mature stage (ZCL), stem in mature stage (ZCS), leaf in tillering stage (ZFL), stem in tillering stage (ZFS), leaf in seedling stage (ZYL), and stem in seeding stage (ZYS).' The roots and buds of ZZ9 (the same parents as ZZ1) were collected at 0d, 1d, 2d, 3d, and 4d for each of the above treatments with three duplicates. Total RNA was extracted from the above samples using RNAprep Pure plant Kit (Tiangen Biotech, Beijing, China) and subsequently used for cDNA library construction. The quality of the cDNA library was assessed on the Agilent Bioanalyzer 2100 system and sequenced on an Illumina Novaseq platform. The original data was quality controlled by Cutadapt, and the quality control data was compared to the ZZ1 genome using HISAT2 v2.1.0 software. Using Cufflinks software, the expression levels of transcripts and genes were quantified through the position information of Mapped Reads on the genome. FPKM (fragments per kilobase

of exon per million fragments mapped) was used as an index to measure the expression level of transcripts.

Line 680-691:

Identification of homologous QTL for smut-resistance

We identified the QTL region for smut resistance on chromosome Sh06 in sugarcane variety R570, spanning approximately 7.74 Mb and containing 512 genes; as a reference, ZZ1 and ROC22 genomes were used as queries. The JCVI (python version MCScan) was employed to locate the chromosomes in ZZ1 and ROC22, each having the maximum number of collinear genes with the QTL region for smut resistance. Subsequently, the identified chromosomes were used as a query. JCVI was utilized to locate the regions with the most densely populated collinear genes as the QTL regions for smut resistance in R570 on homologous chromosomes of ZZ1 and ROC22. JCVI was used to construct collinearity maps between ZZ1 and the smut QTL regions of R570 and ROC22 and employed eggNOG-mapper for functional annotation of genes within the QTL regions for smut resistance in all three varieties.

2- A deeper discussion on the results with a better contextualization and the inclusion of many articles about the complex sugarcane genome;

Response: We added more words to the "Discussions" sections according to your suggestion. The supplemented words are as follows:

Line 339-358:

Despite significant advancements in the haplotype phased genome of two ancestral Saccharum species, which has dramatically advanced the field of sugarcane genomics, they are still unrepresentative of the genomic information of the modern cultivated sugarcane, which possesses many advantageous traits, such as the combination of high sugar content, super abiotic stress resistance, and other exceptional traits. The resolution of these traits is reliant on the complete deciphering of high-quality modern cultivated sugarcane genomes. However, the modern cultivated sugarcane genome is one of the most intricate and challenging worldwide. Over the past two decades, sugarcane genome research pioneers have expended tremendous efforts on the genome of sugarcane cultivars, yet progress has been limited. Unlike allo-polyploids such as wheat, cotton, and Brassica napus, modern cultivated sugarcane originated from crosses between auto-polyploid parents, followed by multiple rounds of backcrossing. Over a long history of breeding, the bloodlines of several parents within the Sugarcane genus have been mixed, resulting in a homo(eo)aneuploid commercially used in asexual reproduction. Approximately 10-20% of the chromosomes in its genome originated from recombination between the parents, and it possesses 8-14 homo(eo)logous copies of most genes. Therefore, the challenges in assembling the genome of modern cultivated sugarcane include (1) distinguishing between homozygous and heterozygous contigs and achieving chromosome-level scaffolding and (2) assembling and scaffolding chromosomes involving recombination events between the original parental lineages.

3- An extended analysis of the genome structure and evolution of this species;

Response: Thanks for your suggestions; we have supplemented the genome structure comparison between ZZ1 and other published sugarcane genomes.

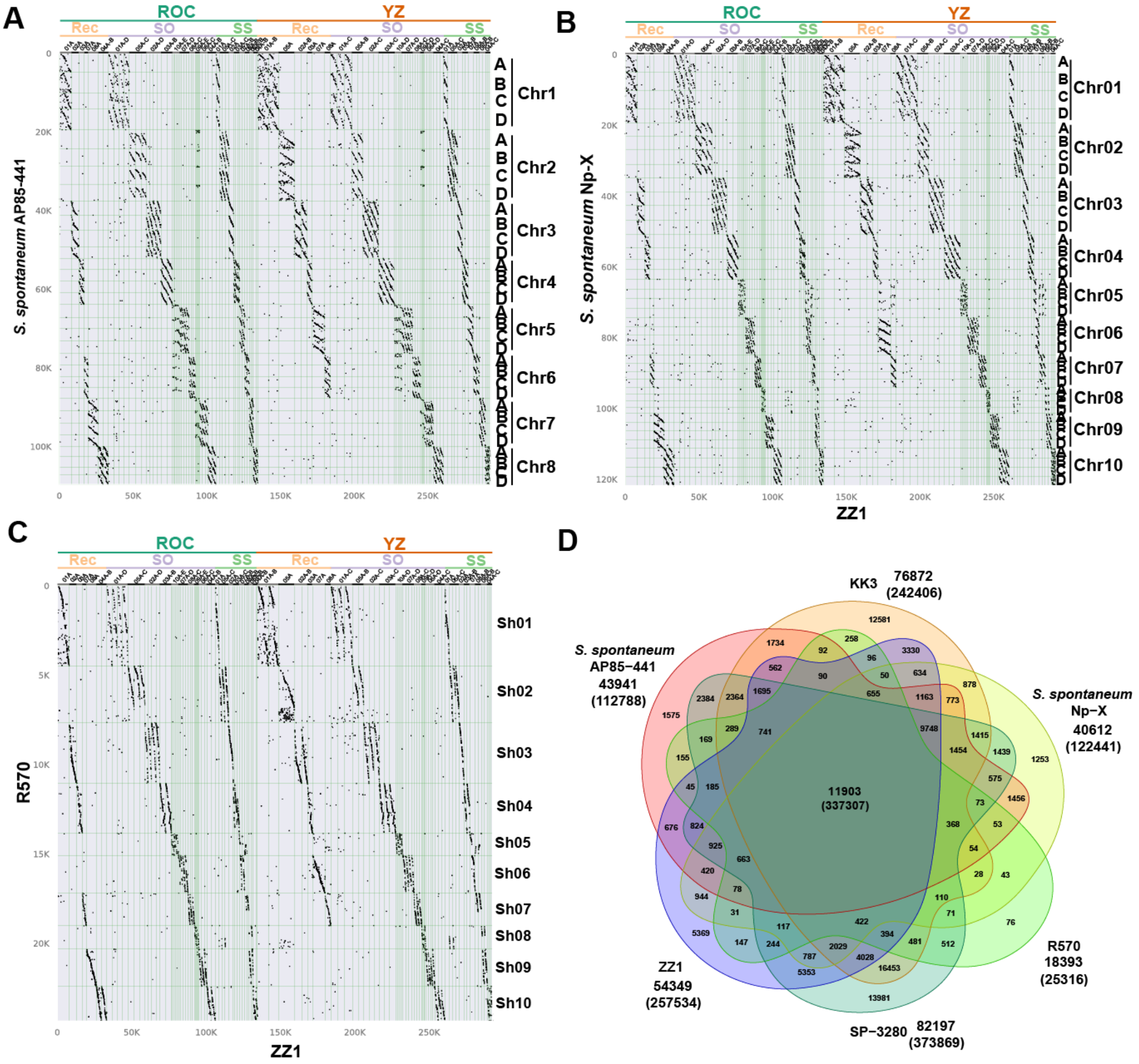

Supplementary Figure 2. The comparison of genome synteny and ortholog gene cluster between ZZ1 and published sugarcane genomes. The genome synteny between ZZ1 and *S. spontaneum* AP85-441(A), *S. spontaneum* Np-X(B), and R570(C). (D) The venn diagram illustrated the ortholog gene clusters among ZZ1 and published sugarcane genomes.

4- Revision of the format of the manuscript including the coherence between text and tables.

Response: We have double-checked and revised the revised version.

Point-By-Point Manuscript Evaluation:

======================================

A. Summary of the key results

The manuscript titled "A chromosomal-scale genome assembly of modern cultivated hybrid sugarcane provides insights into origination and evolution" describes a new genome assembly and analysis of the complex polyploid genome of sugarcane (Saccharum officinarum) including the transcriptomic analysis of several developmental stages. The key results can be summarized as:

• Sequencing, assembly, and annotation of the sugarcane accession ZZ1. The assembly produced has a size of 10.4 Gb. 87% of the assembly was anchored into 114 chromosomes. 66.5% of the genome was annotated as repetitive elements. The genome annotation delivered 257,534 protein-coding gene models. The origin of 68,509 gene models were identified according the three different sub-genomes.
• The cytogenetic characterization of the sugarcane accession ZZ1 described a genome of approx. 9 Gb and 114 chromosomes of which 68 chromosomes derived from S. officinarum, 31 from S. spontaneum, and 15 representing recombination between sub-genomes.
• The authors did not find sub-genome dominance for gene expression, although they found that 7.1% of the gene expression was biased to S. officinarum, and 3.0% to S. spontaneum.
• The pathogen response to Pokkah boeng disease is driven mainly by the S. officinarum, sub-genome rather than the S. spontaneum according to a transcriptomic analysis. Nevertheless, the non-overlapping function of the genes of each sub-genome indicates a possible cooperative response between them.

Response: Thanks for the professional summary!

B. Originality and significance: if not novel, please include reference
----
This manuscript presents a new exciting assembly of the complex sugarcane genome. There have been several genome assemblies of Saccharum spontaneum (Zhang et al., 2018, Zhang et al., 2021) where the origin of this species is discussed. There also some fragmented genome assemblies for S. officinarum (Garsmeur et al., 2018, Souza et al., 2019), but the incompleteness of the assemblies limited the analysis.

Response: Thanks for your suggestion; we have supplemented the words in the Discussion, and the content is as follows:

Line 339-368:

Despite significant advancements in the haplotype phased genome of two ancestral Saccharum species, which has dramatically advanced the field of sugarcane genomics, they are still unrepresentative of the genomic information of the modern cultivated sugarcane, which possesses many advantageous traits, such as the combination of high sugar content, super abiotic stress resistance, and other exceptional traits. The resolution of these traits is reliant on the complete deciphering of high-quality modern cultivated sugarcane genomes. However, the modern cultivated sugarcane genome is one of the most intricate and challenging genomes worldwide. Over the past two decades, sugarcane genome research pioneers have expended tremendous efforts on the genome of sugarcane cultivars, yet progress has been limited. Unlike allo-polyploids such as wheat, cotton, and Brassica napus, modern cultivated sugarcane originated from crosses between auto-polyploid parents, followed by multiple rounds of backcrossing. Over a long history of breeding, the bloodlines of several parents within the Sugarcane genus have been mixed, resulting in a homo(eo)aneuploid commercially used in asexual reproduction. Approximately 10-20% of the chromosomes in its genome originated from recombination between the parents, and it possesses 8-14 homo(eo)logous copies of most genes. Therefore, the challenges in assembling the genome of modern cultivated sugarcane include (1) distinguishing between homozygous and heterozygous

contigs and achieving chromosome-level scaffolding and (2) assembling and scaffolding chromosomes involving recombination events between the original parental lineages.

To overcome the characteristics of polyploidy and extensive recombination among ancestral subgenomes in the modern sugarcane hybrid genomes, we herein proposed a novel 'dimension reduction' assembly strategy, supplemented by a variety of sequencing technologies, to decipher the modern sugarcane hybrid 'ZZ1' genome completely. The quality of this ultra-complex genome, which benefited from innovations in assembly strategies and technological advances, is far superior to the previously published contig-levels SP-3280, draft chromosome-scale KK3, and mosaic R570 monoploid genome, which not only provides a new perspective on the origin and evolution of modern sugarcane hybrid but also helps to analyze the molecular mechanism of excellent traits, which is of great significance for future precision breeding of sugarcane.

C. Data & methodology: validity of approach, quality of data, quality of presentation.

The methodology used looks adequate although there are many missing details in the Material & Methods section that makes difficult a full assessment. For example, the HMW DNA extraction method has not been described in detail (there is only a link to 10X genomics), so it is a gap in the assessment of how the high-quality SMRTbell libraries (30-50 kb) were produced. RNA extraction method has not been specified either, or any of the versions of the program used.

Response: Thanks for your comments. We added more details in the material and methods section. The supplemented content is as following **Line 441-447 and 461-479**:

Illumina short reads sequencing. Genomic DNA was extracted using a QIAGEN DNeasy Plant Mini kit (Qiagen, Hilden, Germany) and subject to library construction with an insert size of 300-500 bp. DNA quality was visually assessed using agarose gel electrophoresis (0.75%), and concentration was estimated using a spectrophotometer (Multiskan Sky Microplate 1510-01307C, Thermo Fisher Scientific, Massachusetts, USA). DNA libraries were sequenced on the Illumina NovaSeq platform with the model of paired-end (PE) 150bp.

RNA extraction, sequencing, and profiling. Spore suspension of *Ssc* (the causative agent of smut) (1×10^6 spores/mL) was inoculated at the growth point of ZZ1, at the young roots and buds, respectively. Inoculated plants were placed in a constant temperature incubator at 28° C in a medium moisturizing culture. Smut group samples were collected at 5d and 20d after inoculation, with water as a control treatment. In addition, the different tissues and different developmental stages were collected from ZZ1, including 'leaf in pre-mature stage (ZBL), stem in pre-mature stage (ZBS), leaf in mature stage (ZCL), stem in mature stage (ZCS), leaf in tillering stage (ZFL), stem in tillering stage (ZFS), leaf in seedling stage (ZYL), and stem in seeding stage (ZYS).' The roots and buds of ZZ9 (the same parents as ZZ1) were collected at 0d, 1d, 2d, 3d, and 4d for each of the above treatments with three duplicates. Total RNA was extracted from the above samples using RNAprep Pure plant Kit (Tiangen Biotech, Beijing, China) and subsequently used for cDNA library construction. The quality of the cDNA library was assessed on the Agilent Bioanalyzer 2100 system and sequenced on an Illumina Novaseq platform. The original data was quality controlled by Cutadapt, and the quality control data was compared to the ZZ1 genome using HISAT2 v2.1.0 software. Using Cufflinks software, the expression levels of transcripts and genes were quantified through the position information of Mapped Reads on the genome. FPKM (fragments per kilobase

of exon per million fragments mapped) was used as an index to measure the expression level of transcripts.

The Hi-C map is not very helpful to identify the possible pseudo-chromosomes. I recommend having in the supplementary a figure with the chromosomes with assigned bins and because there are many of them, split the figure in 6-7 so the readers could inspect the results in more details. In this sense, the authors should also report the number of contacts produced by the Hi-C map.

Respond: Thanks for your suggestion. We supplemented the Hi-C maps that split into 6 parts bases on six pre-assigned groups (ROC-So, ROC-Ss, ROC-Rec, YZ-So, YZ-Ss, YZ-Rec) **(Supplementary Figure 2)** based on the partially Hi-C reads in order to re-run the Hi-C pro in a short period of time. We successfully identified a total of 77,138,022 interaction paired-end reads from the Hi-C map, which accounted for 67.54% of all valid Hi-C reads. Among these, there were 59,906,451 lib valid paired-end reads, representing 77.66% of all interaction paired-end reads. We supplemented this description in the LINE122-123 of revised manuscript.

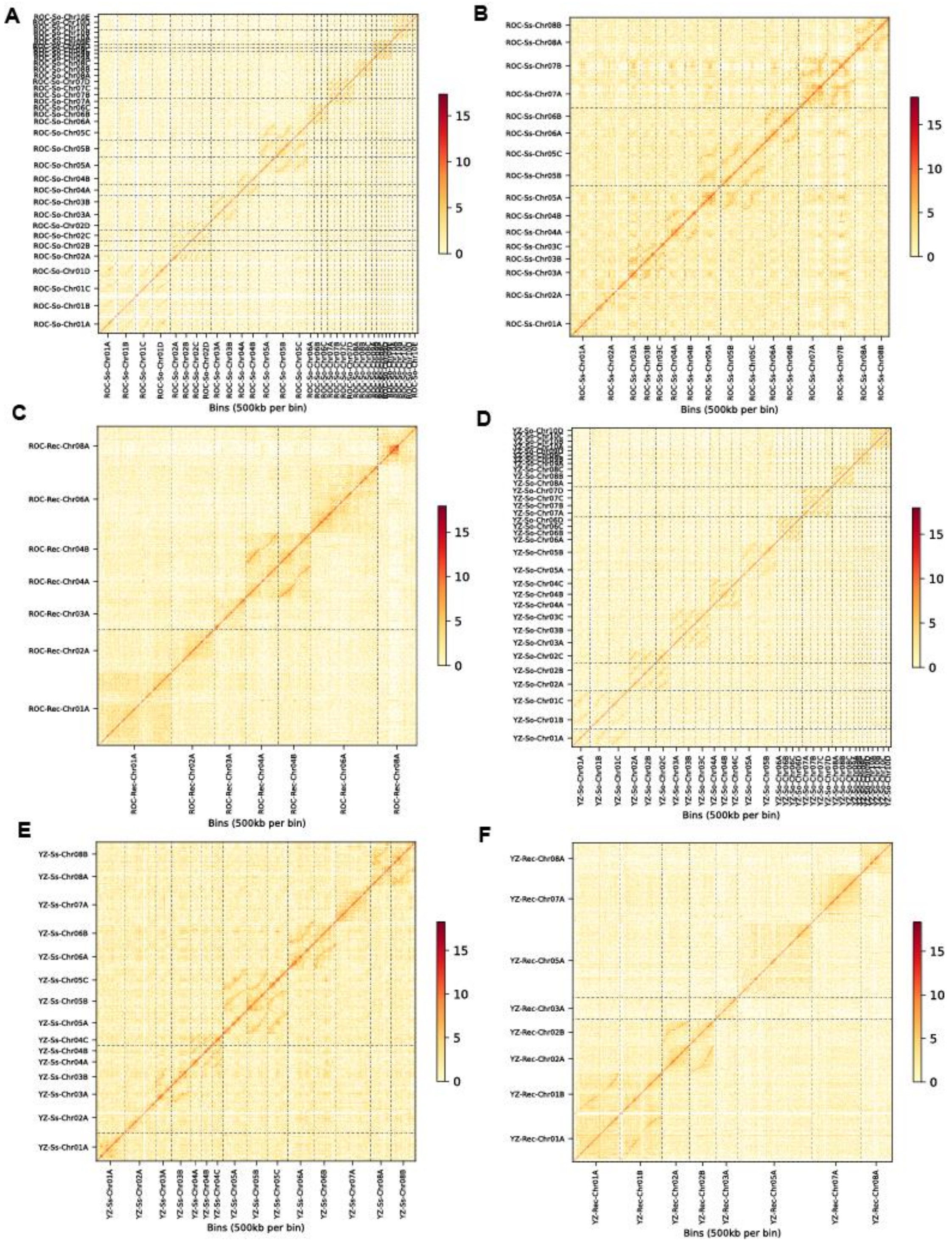

Supplementary Figure 2. Genome-wide analysis of chromatin interactions at 500-kb resolution in six pre-assigned groups among ZZ1 genome. A. ROC-So; B. ROC-Ss; C. ROC-Rec; D. YZ-So; E. YZ-Ss; F. YZ-Rec.

The assessment of the quality of the assembly is partial. The authors have used Merqury but not reported the results about consensus quality and completeness (at least not in the result section). No assessment of the completeness of the LTR elements (e.g., using LTR_retriever). The contamination

screening was performed only by Blast against bacterial genomes, but not, for example, fungi. The use of Blobtools is recommended in this case.

Respond: Thanks for your valuable suggestion. We supplemented the Merqury completeness and quality value (QV) of ZZ1 genome, please see Line 569-576. We successfully identified 716,844 complete LTR elements, spanning a total length of 2.8G. These findings account for 62.91% of all LTR transposable elements. We also calculated the LAI (LTR Assembly Index) to assess the genome assembly. It shows that the ZZ1 genome has a LAI value of 12.27, qualified as a reference genome (Line 140-142).

To address the issue of contaminating sequences and enhance the quality of the ZZ1 genome, we took several steps. Firstly, we downloaded sequences from the NCBI database, including bacterial genomes and plant organelle genomes, to establish a collection of potential contaminating sequences. Subsequently, we employed BLASTN to compare these sequences against the ZZ1 genome and filter out any contaminants. Additionally, we attempted to use the Blobtools process, as you recommended, for further contaminant filtering. To expedite the process, we divided the ZZ1 genome into 50 segments and ran the analysis in parallel. However, due to the substantial size of the dataset and genome, the entire process required several weeks and significant computational resources. Unfortunately, we were unable to complete the process within a reasonable timeframe and had to abandon this approach.

There are some parameters missing in the gene model annotation that could be useful to assess the quality such as the number of single exon gene, length of the gene models, numbers of UTR annotated, number of genes supported by RNA-Seq data… No description of the RNA-Seq analysis, including the number of replicates per sample.

Response: Thanks for your comments. We also supplemented the gene model annotation parameters as you suggested:

Line 156-160: A total of 370,103 protein-coding genes, spanning a combined length of 1235.86 Mb, were annotated by protein-homology-predicted and RNA-seq-aligned methods, of which 92.14% (341,040) could be successfully validated by RNA-seq reads. Out of all the annotated genes, 30.03% (111,167) consisted solely of a single exon, while 14.26% (52,797) and 15.75% (58,308) genes had 5’UTR and 3’UTR annotations.

We supplemented the description of the RNA-Seq analysis as following:

Line 465-479: The different tissues and different developmental stages were collected from ZZ1, including 'leaf in pre-mature stage (ZBL), stem in pre-mature stage (ZBS), leaf in mature stage (ZCL), stem in mature stage (ZCS), leaf in tillering stage (ZFL), stem in tillering stage (ZFS), leaf in seedling stage (ZYL), and stem in seeding stage (ZYS).' The roots and buds of ZZ9 (the same parents as ZZ1) were collected at 0d, 1d, 2d, 3d, and 4d for each of the above treatments with three duplicates. Total RNA was extracted from the above samples using RNAprep Pure plant Kit (Tiangen Biotech, Beijing, China) and subsequently used for cDNA library construction. The quality of the cDNA library was assessed on the Agilent Bioanalyzer 2100 system and sequenced on an Illumina Novaseq platform. The original data was quality controlled by Cutadapt, and the quality control data was compared to the ZZ1 genome using HISAT2 v2.1.0 software. Using Cufflinks software, the expression levels of transcripts and genes were quantified through the

position information of Mapped Reads on the genome. FPKM (fragments per kilobase of exon per million fragments mapped) was used as an index to measure the expression level of transcripts.

The quality of the genome assembly should be described using the standards of the Earth Biogenome Project (https://www.earthbiogenome.org/assembly-standards).

Respond: Thanks for your suggestion. We sincerely appreciate your interest in the quality of our genome assembly and value your valuable suggestions. We made an effort to utilize the standards outlined in the Earth Biogenome Project (https://www.earthbiogenome.org/assembly-standards) that you recommended. However, it appears that the provided URL is currently invalid.

D. Appropriate use of statistics and treatment of uncertainties

----

Yes, in some extension (see previous section).

Response: Thanks for your comments. We have revised in the revised version.

E. Conclusions: robustness, validity, reliability

The conclusions are valid according to the results presented. However, the lack of details makes difficult a full assessment (for example, for the expression analysis, it needs to be assumed that the authors used three independent replicates of plants challenged by the pathogen and with clear phenotypes of the infection… none of these details are in the material and methods).

Response: Thanks for your comments. We added more details in the material and methods section. The supplemented content is as follows: Line 461-479.

RNA extraction, sequencing, and profiling. Spore suspension of *Ssc* (the causative agent of smut) (1×10^{6} spores/mL) was inoculated at the growth point of ZZ1, at the young roots and buds, respectively. Inoculated plants were placed in a constant temperature incubator at 28° C in a medium moisturizing culture. Smut group samples were collected at 5d and 20d after inoculation, with water as a control treatment. In addition, the different tissues and different developmental stages were collected from ZZ1, including 'leaf in pre-mature stage (ZBL), stem in pre-mature stage (ZBS), leaf in mature stage (ZCL), stem in mature stage (ZCS), leaf in tillering stage (ZFL), stem in tillering stage (ZFS), leaf in seedling stage (ZYL), and stem in seeding stage (ZYS).' The roots and buds of ZZ9 (the same parents as ZZ1) were collected at 0d, 1d, 2d, 3d, and 4d for each of the above treatments with three duplicates. Total RNA was extracted from the above samples using RNAprep Pure plant Kit (Tiangen Biotech, Beijing, China) and subsequently used for cDNA library construction. The quality of the cDNA library was assessed on the Agilent Bioanalyzer 2100 system and sequenced on an Illumina Novaseq platform. The original data was quality controlled by Cutadapt, and the quality control data was compared to the ZZ1 genome using HISAT2 v2.1.0 software. Using Cufflinks software, the expression levels of transcripts and genes were quantified through the position information of Mapped Reads on the genome. FPKM (fragments per kilobase of exon per million fragments mapped) was used as an index to measure the expression level of transcripts.

F. Suggested improvements: experiments, data for possible revision.

The manuscript lacks important details in the material and method section.

Response: Thanks for your comments. We added more details in the material and methods section. The supplemented content is as follows: Line 441-447, 461-479, and 680-691.

.

The Discussion also should be improved. There is no extensive discussion of the polyploid nature of the genome in the context of origin (e.g., no dating or comparison with the results of the Saccharum spontaneum articles (Zhang et al., 2018; Zhang et al., 2021)). The Discussion is more a summary rather than a contextualization in which different results are compared with previously published results driving to new hypothesis and insights.

Response: Thank you for your professional and constructive comments. We have incorporated your feedback into the revised manuscript, explicitly focusing on the discussion section. In this revision, we have included a comprehensive analysis of the complexity and assembly challenges encountered during sequencing the ZZ1 genome. Additionally, we have compared our findings with previously published papers on the sugarcane genome, highlighting similarities and differences. Furthermore, we have dedicated a section to discuss the difficulties faced during this study, shedding light on the intricacies of the research process. We have also emphasized the notable findings and breakthroughs achieved through our analysis. We sincerely appreciate your valuable input, which has immensely enriched the discussion section of our revised manuscript. Here are the detailed updates made to the discussion section in the revised manuscript:

Line 339-368:

Despite significant advancements in the haplotype phased genome of two ancestral Saccharum species, it is still unclear how the two ancestor species' descent created the high sugar content, super abiotic stress resistance, and other excellent traits of modern hybrid cultivated varieties, which all depend on the complete decipher of high-quality genomes, and has dramatically advanced the field of sugarcane genomics; they still need to be representative of the genomic information of the modern cultivated sugarcane, which possesses many advantageous traits, such as the combination of high sugar content, super abiotic stress resistance, and other exceptional traits. The resolution of these traits is reliant on the complete deciphering of high-quality modern cultivated sugarcane genomes. However, the modern cultivated sugarcane genome is one of the most intricate and challenging worldwide. Over the past two decades, sugarcane genome research pioneers have expended tremendous efforts on the genome of sugarcane cultivars, yet progress has been limited. Unlike allo-polyploids such as wheat, cotton, and Brassica napus, modern cultivated sugarcane originated from crosses between auto-polyploid parents, followed by multiple rounds of backcrossing. Over a long history of breeding, the bloodlines of several parents within the Sugarcane genus have been mixed, resulting in a homo(eo)aneuploid commercially used in asexual reproduction. Approximately 10-20% of the chromosomes in its genome originated from recombination between the parents, and it possesses 8-14 homo(eo)logous copies of most genes. Therefore, the challenges in assembling the genome of modern cultivated sugarcane include (1) distinguishing between homozygous and

heterozygous contigs and achieving chromosome-level scaffolding and (2) assembling and scaffolding chromosomes involving recombination events between the original parental lineages.

To overcome the characteristics of polyploidy and extensive recombination among ancestral subgenomes in the modern hybrid sugarcane hybrid cultivar genomes, we herein proposed a novel 'dimension reduction' assembly strategy, supplemented by a variety of sequencing technologies, to completely decipher the modern hybrid sugarcane cultivar hybrid 'ZZ1' genome. Theis quality of this ultra-complex genome, which benefited from innovations in assembly strategies and technological advances, is far superior to the previously published contig-levels SP-3280, draft chromosome-scale KK3, and mosaic R570 monoploid genome, which not only provides a new perspective on the origin and evolution of modern sugarcane hybrid cultivated sugarcane but also helps to analyze the molecular mechanism of excellent traits, which is of great significance for future precision breeding of sugarcane.

Line 393-403:

The high-quality genome of ZZ1 demonstrates its chromosome origin and recombination in high resolution, expanding our knowledge of its chromosomes from the quantitative level to the specific sequence level. Notably, previous studies have indicated the presence of three chromosome bases, namely 8, 9, and 10, in S. spontaneum. However, it has remained uncertain which bases were used as parents in early sugarcane hybrid cultivars. Our genomic evidence now demonstrates that the ancestral parental chromosome base contributing to S. spontaneum consanguinity in ZZ1 is chromosome base 8, excluding bases 9 and 10. This conclusion is drawn from the observation that the S. spontaneum pedigree of the ZZ1 genome lacks intact ancestral chromosomes from S. spontaneum, specifically Chr05 and Chr08 homologous groups. This finding may also play a significant role in explaining the formation of aneuploids in hybrid sugarcane during the breeding process.

The authors did an excellent characterization of the origin of the different sub-genomes. Nevertheless, I think that there are several pieces that it could be interesting to explore about the history of this complex genome:

• Tracing of the maternal inherence of the organelle genomes through the different rounds of hybridization and polyploidization. The authors did not mention anything about the chloroplast and mitochondria origin of the ZZ1 accession. The suppl. figure 1 states ROC1 as the maternal donor but it will be interesting to know if they derived from S. robustum or S. officinarum.

Response: Thanks. It is a fascinating topic. However, this study concentrated primarily on the nuclear genome and had no intention of isolating and assembling the chloroplast or mitochondrial genomes. Future investigations on sugarcane evolution could take advantage of this approach. ROC25 is the fifth generation of *S. robustum*.

• Dating of speciation and hybridization events. The authors did estimate the divergency between the different sub-genomes using the synonymous substitution (Ks) of the ortholog pairs of SO-ZZSO, SS-ZZSS, ZZSS-ZZSO, and SO-SS. It will add value to the results and the Discussion to see some age estimation associated to this Ks ratio values. The authors could even use the Miscanthus genomes (Zhang et al. 2021) to date the possible divergency dates between the different sub-genomes (Miscanthus- Saccharum split dated as 7.9 MYA according https://timetree.org/).

Response: Thank you for your highly professional advice. We have thoroughly analyzed the Ks values between the two subgenomes of ZZ1 and Miscanthus. The calculated Ks values for ZZSO-Miscanthus and ZZSS-Miscanthus are 0.0763 and 0.0764, respectively. These values indicate a similar level of divergence between the subgenomes, which aligns with the comparable divergence times observed. The divergence between Miscanthus and ZZ1 appears to be significantly greater compared to the divergence between the subgenomes.

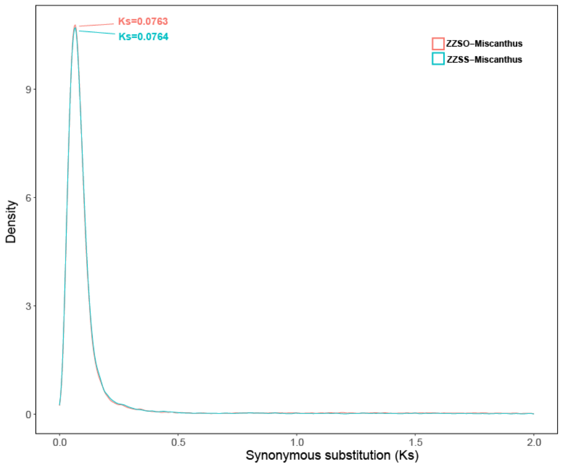

Supplementary Figure 7. The comparison of synonymous substitution (Ks) between subgenomes of ZZ1 and Miscanthus.

• Homologous exchange transposition (HET) characterization. There is not an extensive analysis of the regions of the genome that present HET. It could be interesting to have a deeper look of this part with some questions (for example, are the HET regions rich in TEs favoring the non-reciprocal exchanges?).

Response: Thank you for your excellent suggestion. We have conducted a thorough analysis comparing the transposon content of ZZSO, ZZSS, and the recombination-prone region (Rec) in the ZZ1 genome. Our findings indicate a significantly higher transposon content in the ZZSO region compared to ZZSS and Rec. However, it is essential to note that the transposon content in the ZZSS and Rec regions is relatively similar. This leads us to hypothesize that several other factors influence homologous recombination exchange, which extends beyond the scope of transposons alone.

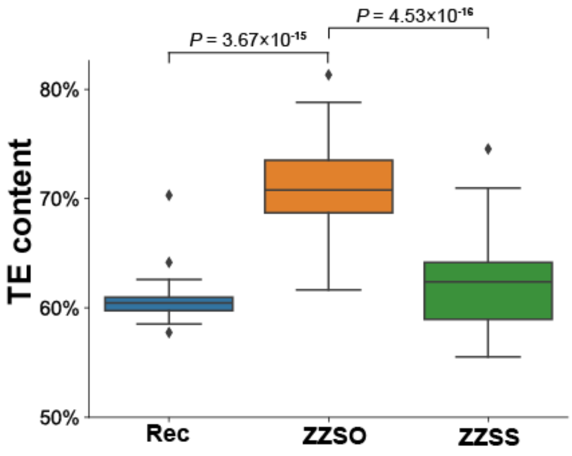

Supplementary Figure 6. The comparison of TE content between the Rec, ZZSO, ZZSS region of ZZ1 genome. Significance was tested with t-test. In the box plots, central line: median values; bounds of the box: 25th and 75th percentile.

The manuscript needs a format revision. For example, the first time that you introduce a species name, it needs to be used as complete name (e.g., Saccharum officinarum). Then, it can be abbreviated using only the first letter of the genus (as S. officinarum). In the line 182, the species S. officinarum is mistyped as S. officianrum and in the line 210 as Sacchuarm. In some paragraphs, the authors use the species names (S. spontaneum and S. officinarum) while in other ones they use just an abbreviation (SS or SO). The same criteria should be used for the whole text. It also needs a consistency revision between the numbers presented in the text and the tables. For example, in the line 141 is described that 257,534 protein-coding gene models were identified. Nevertheless, in the table 1, this number goes to 370,103.

Response: Thank you for your comments. We thoroughly reviewed them in the revised manuscript. Regarding your question, the accurate number of predicted gene models should be 370,103 instead of 257,534, which is the older version of the genome annotation. We have revised the number and related results.

G. References: appropriate credit to previous work?

Yes, in some extend, but I found some references missing like the one for the reference genome S. officinarum LA-purple.

Response: Thank you for pointing out this issue. The *S. officinarum* LA-purple genome, which is currently being submitted by our collaborator, has been indicated as "unpublished" throughout the revised manuscript.

H. Clarity and context: lucidity of abstract/summary, appropriateness of abstract, introduction and conclusions.

The abstract is adequate, and clear.
Response: Thanks.
Revised by Aureliano Bombarely on Oct. 31st, 2023.

Reviewer #3 (Remarks to the Author):

In the manuscript entitled “A chromosomal-scale genome assembly of modern cultivated hybrid sugarcane provides insights into origination and evolution,” the authors describe a haplotype resolved chromosome-scale assembly of the ZZ1 cultivar of sugarcane. The authors describe how they assembled the highly complex ZZ1 genomes using long and short reads couple to HiC. In the abstract the authors claimed they developed new methods (“…series of bioinformatics approaches we developed…”), but it was unclear from the description that any new/novel assembly techniques were used in the process. The authors then go on to look at homoeolog expression

dominance and conclude there is none in ZZ1. In addition, the authors look at expression after treatment with the fusarium pest Pokkah boeng, and report that it may be a combination of the subgenomes that provides resistance (based on expression). The manuscript reads much like a genome announcement and only addresses the origin and evolution of the sugarcane genome through the comparison and expression of alleles. Since this is primarily a manuscript about an improved sugarcane genome assembly, the genome needs to be available to the reviewers to assess; impossible to review the content otherwise. Also, the authors do very little to no comparison with the sugarcane genome assemblies that are currently available.

Response: Thank you for your comments. We have further improved the material and methods section to clarify our newly developed methods for highly complex genome assembly. Additionally, we have incorporated new genomic comparison analysis results into the revised version of the manuscript. As per your suggestions, we have included the following in the revised manuscript: (1) A comparison of the assembly characteristics of the ZZ1 genome with the published genomes of the foundational sugarcane species and the sugarcane cultivar genomes. (2) A comparison of synteny between ZZ1 and the published sugarcane genomes. (3)The comparison of the set of genes that exhibit direct homology to each other, shedding light on potential evolutionary relationships and functional similarities. Furthermore, we have incorporated an analysis of Smut resistance in sugarcane, providing insights into the genetic factors associated with this trait. (4) Data availability. Relevant data supporting the findings of this study are available in this article and its Supplementary Information files. The the raw data, genome assemblies, and annotation data generated in this study have been deposited in the China National Center for Bioinformation Genome Warehouse under accession code GWHEQVP00000000. The SP80-3280 genome data were downloaded from NCBI under accession number GCA_009173535.1; KK3 were downloaded from NCBI under accession number JALQSO000000000; R570 were downloaded from EMBL under accession number ERZ654945; AP85-441 were downloaded from NCBI under accession number QVOL00000000; NP-X were deposited into the Sequence Read Archive (under BioProject accession PRJNA721787); LA-Purple were available from NCBI with the same accession number and under Bioproject accession PRJNA744175; Rice, Sorghum and maize information was collected from the article49-51.

Supplementary Table 7. Comparison of assembly parameters of ZZ1 with previously published sugarcane genomes.

	Sugarcane cultivars (homo(eo)aneuploid)				Sugarcane wild species	
	ZZ1	R570	KK3	SP80-3280	*S. spontaneum* Np-X	*S. spontaneum* AP85-441
**Total of scaffolding Chromosomes**	114	10	56	-	40	32
**Size of Assembly (Gb)**	10.400	0.382	7.0	4.260	2.760	3.130
**Contig N50 (Kb)**	125	-	83	13	381	45
**No. of annotated gene (alleles)**	257,534	25,316	242,406	373,869	122,441	112,788
**BUSCO (%)**	99.7	-	-	90.9	96.46	97.01

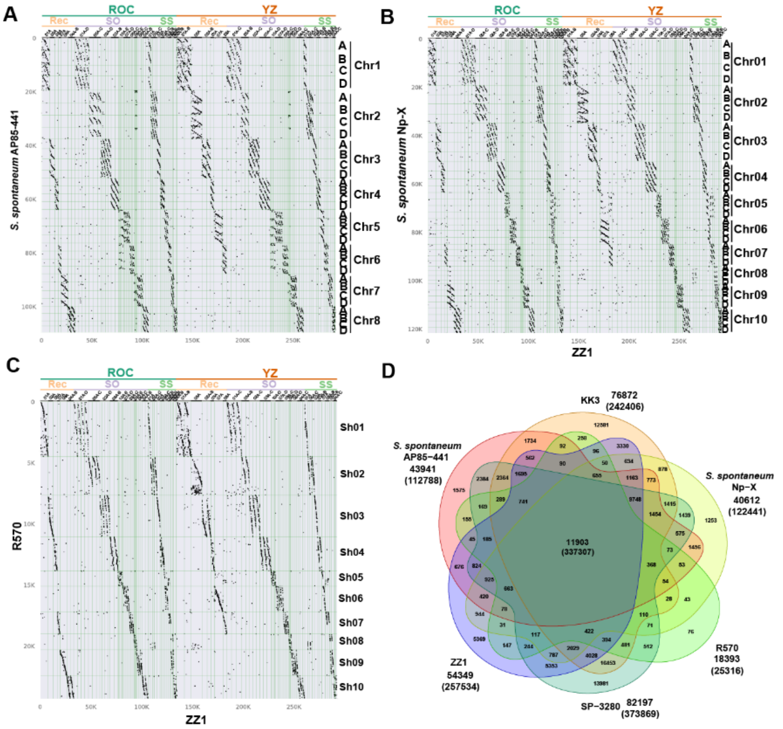

Supplementary Figure 3. The comparison of genome synteny and ortholog gene cluster between ZZ1 and published sugarcane genomes. The genome synteny between ZZ1 and *S. spontaneum* AP85-441(A), *S. spontaneum* Np-X(B), and R570(C). (D) The Venn diagram illustrated the ortholog gene clusters among ZZ1 and published sugarcane genomes.

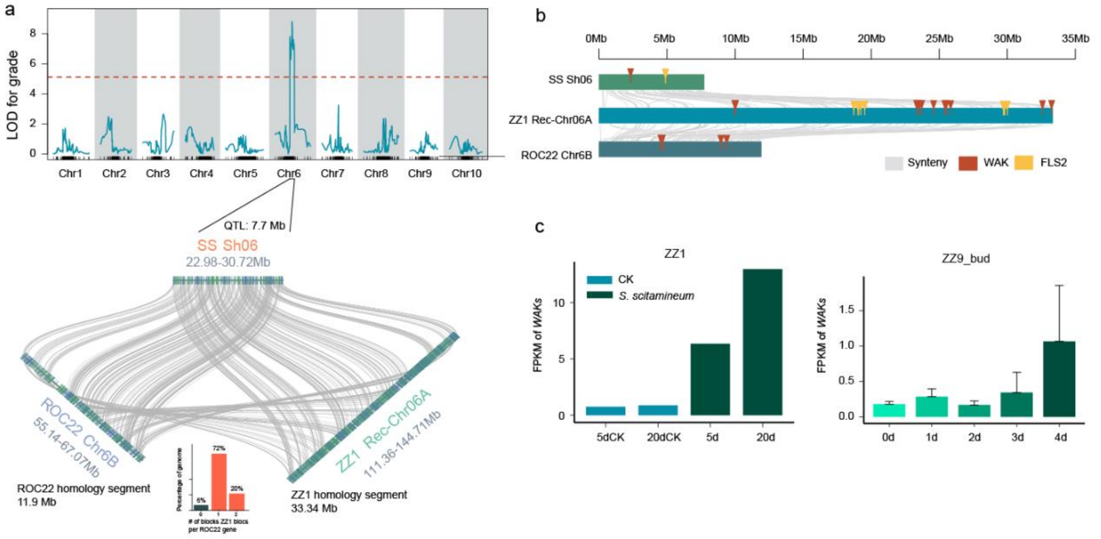

Figure 5. The gene family associated with resistance in modern sugarcane breeding. (a). Using the R570 QTL region (7.74Mb) for smut resistance as a reference, QTL regions for smut resistance were identified in ZZ1 and ROC22, with gene collinearity shown within the QTL region connected by gray curved lines. (b). Syntenic distribution of resistance-related gene families within the homologous region. Synteny is represented by gray curved lines, the WAK gene family is indicated in deep brown, and the FLS2 gene family is represented in yellow. (c).Expression patterns of the WAK gene family. Depicted using bar graphs. The x-axis represents different treatment times for

root and bud. In contrast, the y-axis represents FPKM (fragments per kilobase of transcript per million mapped reads).

Below are small comments concerning grammar content and writing.

If you are going to use “Rec” to represent the recombined chromosomes, then use it consistently throughout.

Response: Thank you very much for pointing out this mistake to us. We have thoroughly proofread it in the revised manuscript.

Please use ZZ1 or ZZ consistently throughout as the name of the cultivar.

Response: Thank you very much for pointing out this mistake for us. ZZ1 is the exact name of this sugarcane hybrid cultivar; we have thoroughly proofread it in the revised manuscript.

Would be great to add a bit in the introduction about Pokkah boeng (and disease resistance in sugarcane) in the introduction.

Response: Thank you for your excellent suggestion. We have considered it and made the necessary revisions to the INTRODUCTION section of the revised draft. We have included a detailed description of Pokkah boeng, highlighting its detrimental effects on sugarcane. These additions provide a comprehensive overview of the topic, and the supplementary content as follows:

Line 69-76:

Smut, one of the most severe diseases, caused 20%~30% yield and sugar losses in China. Pokkah boeng disease (PBD), a century-old disease worldwide, is becoming more serious in China due to the overuse of nitrogen fertilizer and the warming of the climate. Herein, we present a haplotype-resolved, contiguous, chromosome-scale de novo assembly of the hybrid sugarcane genome, ZZ1, which is extensively grown in Guangxi, China, for its resistance to sugarcane smut and susceptible to pokkah boeng disease PBD. Our work aims to deepen our understanding of genome architecture and expedite sugarcane improvement efforts on resistance against sugarcane smut and pokkah boeng disease.

Line 45 “….leading to a colossal genome size of ~10 Gb.”

It is large but not “colossal;” gymnosoperms and above 40 Gb is colossal-this is just a large genome for a plant.

Response: Thank you for your suggestions. We have great insights into complex genomes, have adopted the suggestions, and have revised the manuscript.

Lines 133-135: “The comprehensive approach was used to detect allele genes within the ultra-complex genome based on the monoploid genomes and annotated genes of two ancestors (see Method).”

Sentence does not make sense.

Response: Thanks for your comment; we have rewritten this sentence as follows:
"The comprehensive approach was employed to identify allele genes within this ultra-complex genome relied on the monoploid genome-annotated gene sets from two sugarcane foundation species as reference."

In the section from lines 156-182, the authors use the word "decents;" do the authors mean descendants? It could also be used to mean descent (trajectory) but that does not fit well in the context of what is being said.

Response: Thank you for pointing out this issue. Indeed, using the word "descents" may not be suitable in this context. After careful consideration, we have used the term "pedigree" to convey the meaning accurately. We have already replaced all instances of "descent" with "pedigree" in the revised manuscript.

Lines 211-213: "Sugar transporter family: A total of 130 genes, including 1,166 alleles, likely belong to the members of the sugar transporter superfamily consisting of PLT, VGT, SFP, TMT, STP, pGlcT, INT, SUT, and SWEET subfamily." Spell out

Response: Thanks for your comment! We added these gene names in the revised manuscript.

Line 245-249:

polyol/monosaccharide transporters (PLT), vacuolar glucose transporter (VGT), early-responsive to dehydration protein (SFP), tonoplast monosaccharide transporters (TMT), sugar transporter protein (STP), plastidic glucose transporter (pGlcT), inositol transporter (INT), sucrose transporter (SUT), and the Sugars Will Eventually be Exported Transporters (SWEET).

Line 219: "…and their reconstruction in the leaf of the different development stage…"
Not sure what this means; please clarify.

Response: Thanks for pointing this out to us. We have revised this sentence in the revised version. The rewritten content is as follows:

Line 253-257:

"The expression profile of these alleles showed that the alleles derived from *S. officinarum* were significantly more abundant than those derived from *S. spontaneum* across the different stages of leaf reconstruction (Figure 4b), indicating that the genetic basis for sugar transport in ZZ1 is significantly contributed by *So*."

Lines 221-223: "In addition, the PLT, TMT, and SWEET families had more gene members than S. spontaneum, S. officinarum, and their relative species, suggesting that gene expansion occurred in these three families during the breeding of ZZ."

Response: Thank you for pointing out this mistake; we have rewritten this sentence in the revised version. The rewritten sentence is as follows:

Line 257-259:

“In addition, ZZ1 has more members in the PLT, TMT, and SWEET families than Ss, So, and their relative species, suggesting that gene expansion occurred in these three families during the breeding of ZZ1.”

Lines 225-231: The section on NBS has very little content.

Response: Thank you for your comment. We have made several revisions to the text based on your suggestions. Firstly, we have provided additional descriptions for NBS to enhance the readers' understanding. Furthermore, we have included more information regarding disease resistance, explicitly addressing smut. Additionally, we have rephrased the section "The lineages dominate expression to participate in Pokkah boeng." Please refer to the revised version for more detailed information.

Line 240: “lineages showed dominant expression (LDE)”
Does LDE stand for Lineage Dominant Expression? If so they this sentence needs to be reconstructed to make that clear.

Response: Thanks for your suggestion! We have revised the resubmitted version (Line 295- 299).

Line 255: “…genes that showed up-regulated in SO…”
Maybe should read, “…genes that were up-regulated in SO…”

Response: Thank you so much for helping us point out this mistake. We have corrected it in the revised version (Line 313-316).

Reviewers' Comments:

Reviewer #1:
Remarks to the Author:
I am satisfied that the issues raised in the first submission have been adequately addressed.

Reviewer #2:
Remarks to the Author:
Overall evaluation of the reviewed version
==
The authors have resolved all the comments and concerns risen during my first revision. They have completed the material and method so the methodology can be assessed and followed, if necessary, in most of the cases. Nevertheless, there are still some incomplete information. For example, it is still not clear how the QTL analysis was performed (section: Identification of homologous QTL for smut-resistance). The manuscript has a reference about the article "A mosaic monoploid reference sequence for the highly complex genome of sugarcane" but in this article is not described how the smut resistance region was identified, so I am not sure if it is the wrong citation. The discussion has been extended, but there is missing the contextualization comparing the results produced in this manuscript with other complex genomes, citing sources when needed. Beyond these small concerns, the manuscript is interesting and bring new exciting results about this very complex genome.

Revised by Aureliano Bombarely on Feb. 1st, 2024

Reviewer #3:
Remarks to the Author:
In the revised manuscript the authors have enhanced the clarity of several points and integrated comparative findings that provide essential context for interpretation. The manuscript presents a compelling model for future analyses of sugarcane. The outcomes are poised to be immediately valuable for contemporary studies, and the paper is poised to hold enduring significance in advancing our understanding of hybrid sugarcane genome organization and evolution. Many of my initial questions and concerns have been effectively addressed; following are minor suggestions:
Line 345: Replace "Sugarcane genus" with "Saccharum genus."
Lines 421/422: The text appears redundant in comparison to lines 266/267 and can be omitted.
Lines 875/876: Adjust "x-axis" to "y-axis" for clarity in presentation.

REVIEWERS' COMMENTS

Reviewer #1 (Remarks to the Author):

I am satisfied that the issues raised in the first submission have been adequately addressed.
Response: Thank you for your recognition of our work and for your helpful suggestions, which have helped improve our manuscript.

Reviewer #2 (Remarks to the Author):

Overall evaluation of the reviewed version
==
The authors have resolved all the comments and concerns risen during my first revision. They have completed the material and method so the methodology can be assessed and followed, if necessary, in most of the cases. Nevertheless, there are still some incomplete information. For example, it is still not clear how the QTL analysis was performed (section: Identification of homologous QTL for smut-resistance).
Response: Thank you for your highly professional suggestions. We added more details of QTL analysis for smut-resistance in the methods section. The supplemented content is as following:

Line 672-692:

We screened the large F1 population (c. 17000 individual clones) of parental (ROC25 x YZ89-7) and constructed a smut-resistant/susceptible subpopulation. After field evaluation for three consecutive years, a total of 401 clones were verified, and each clone was assigned a resistance value (from 1 to 8, 1 representing the most resistant and 8 representing the most susceptible). Genotyping by Sequencing (GBS) technology was employed to sequence ROC25, YZ89-7, and 236 clones from the F1 population. This approach yielded 12,975,602 SNP sites, from which 1,196 bin markers were derived after screening and filtering. Utilizing R570[18] as the reference genome, these bin markers were utilized to construct a high-density genetic map of sugarcane, spanning a total length of 701.855 cM, with individual linkage groups varying from 59.855 cM to 81.681 cM. Subsequently, simplified genome sequencing was conducted on 220 individuals exhibiting diverse resistance levels in field conditions. Based on the constructing the high-density genetic map for sugarcane, permutation calculation used disease grade as phenotypic data and determined the screening threshold to be 7.119. Next, QTL positioning analysis was conducted using R/QTL v1.39 software, employing Composite Interval Mapping (CIM) to depict a curve graph based on the LOD value of each site. Results indicated the initial location of the smut QTL on the Sh06 chromosome of the R570 genome. The QTL confidence interval, spanning 5 cM from both ends of the peak and declining by 1.5 LOD values (Figure 5a), revealed a phenotypic variance explanation of 22.74%. The length of the confidence interval measured 2.967 cM, spanning approximately 7797.632 Kb and

containing 512 genes within the QTL interval.

The manuscript has a reference about the article “A mosaic monoploid reference sequence for the highly complex genome of sugarcane” but in this article is not described how the smut resistance region was identified, so I am not sure if it is the wrong citation.
Response: Thank you for pointing out this mistake, We have removed this wrong citation in the revised manuscript.

The discussion has been extended, but there is missing the contextualization comparing the results produced in this manuscript with other complex genomes, citing sources when needed. Beyond these small concerns, the manuscript is interesting and bring new exciting results about this very complex genome.
Response: Thank you very much for helping us point out these issues, which do cause confusion and distress to readers, and we have further added content pointers and literature citations in the revised manuscript.

Revised by Aureliano Bombarely on Feb. 1st, 2024

Reviewer #3 (Remarks to the Author):

In the revised manuscript the authors have enhanced the clarity of several points and integrated comparative findings that provide essential context for interpretation. The manuscript presents a compelling model for future analyses of sugarcane. The outcomes are poised to be immediately valuable for contemporary studies, and the paper is poised to hold enduring significance in advancing our understanding of hybrid sugarcane genome organization and evolution. Many of my initial questions and concerns have been effectively addressed; following are minor suggestions:
Line 345: Replace "Sugarcane genus" with "Saccharum genus."
Lines 421/422: The text appears redundant in comparison to lines 266/267 and can be omitted.
Lines 875/876: Adjust "x-axis" to "y-axis" for clarity in presentation.
Response: Thanks for your professional summary and comments. We have revised the minor detail and Figure 2B in the revised version.

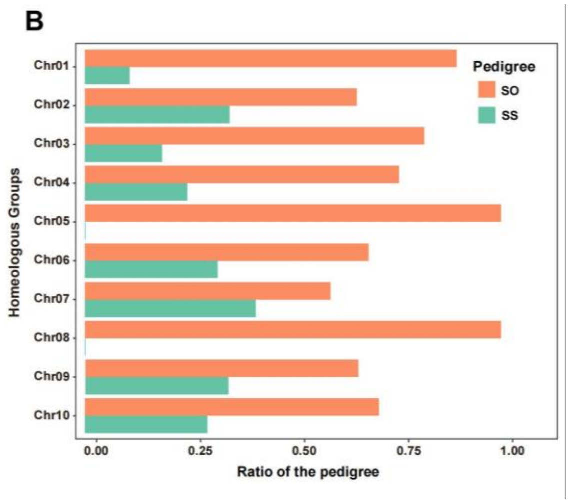

Figure 2. The pedigree identification and analysis of ZZ1. (B) The histogram displayed the ratio of the pedigree of S. officinarum and S. spontaneum in each homologous group. The x-axis means the ratio of the pedigree, and the y-axis represents the homeologous groups from Chr1 to Chr10.
